# Cognitive and Psychological Symptoms in Post-COVID-19 Condition: A Systematic Review of Structural and Functional Neuroimaging, Neurophysiology, and Intervention Studies

**DOI:** 10.1016/j.arrct.2025.100461

**Published:** 2025-05-09

**Authors:** Eva Pettemeridou, Maria Loizidou, Jelena Trajkovic, Maria Constantinou, Stefanie De Smet, Chris Baeken, Alexander T. Sack, Steven C.R. Williams, Fofi Constantinidou

**Affiliations:** a Center for Applied Neuroscience, University of Cyprus, Nicosia, Cyprus; b KIOS Research and Innovation Center of Excellence, University of Cyprus, Nicosia, Cyprus; c Department of Psychology, University of Cyprus, Nicosia, Cyprus; d Department of Head and Skin, Ghent Experimental Psychiatry (GHEP) Laboratory, Ghent University, Ghent, Belgium; e Department of Psychiatry, Ghent University Hospital, Ghent, Belgium; f Department of Psychiatry, Vrije Universiteit Brussel (VUB), Universitair Ziekenhuis Brussel (UZ Brussel), Brussels, Belgium.; g Department of Electrical Engineering, Eindhoven University of Technology, Eindhoven, The Netherlands; h Department of Cognitive Neuroscience, Faculty of Psychology and Neuroscience, Maastricht University, Maastricht, The Netherlands; i Department of Head and Skin, Psychiatry and Medical Psychology, Ghent University Hospital, Ghent, Belgium; j Department of Electrical Engineering, Eindhoven University of Technology, Eindhoven, The Netherlands.; k Department of Neuroimaging, Institute of Psychiatry, Psychology and Neuroscience, King’s College London, London, UK

**Keywords:** Brain injuries, Cognitive rehabilitation, EEG, Functional connectivity, Long COVID, MRI, Neuropsychological impairment, Noninvasive brain stimulation, Rehabilitation, White matter changes

## Abstract

**Objective:**

To investigate the structural, functional, and neurophysiological brain changes associated with post-COVID-19 condition (PCC)-related cognitive and psychological issues and evaluate the efficacy of noninvasive brain stimulation (NIBS) and cognitive rehabilitation interventions.

**Data Sources:**

Electronic databases, including Web of Science, PubMed, and Embase, were systematically searched for articles published before February 1, 2025, using terms such as “post-COVID-19 condition,” “cognitive dysfunction,” “brain changes,” “noninvasive brain stimulation,” and “cognitive rehabilitation.” Language was restricted to English, and only studies involving human participants were included.

**Study Selection:**

Studies with human participants aged ≥18 years diagnosed with PCC, employing magnetic resonance imaging, functional magnetic resonance imaging, positron emission tomography, and electroencephalography, and interventions such as NIBS and cognitive rehabilitation were included. Articles were selected through independent review by multiple authors, with consensus resolving discrepancies. Of the 123 studies initially identified, 78 met the inclusion criteria.

**Data Extraction:**

Data on participant demographics, methodologies, neurophysiological changes, and intervention outcomes were extracted by 2 independent reviewers using predefined guidelines. Study quality was assessed using the Newcastle-Ottawa Scale and Critical Appraisal Skills Program tools.

**Data Synthesis:**

Seventy-eight studies with over 5900 participants met the inclusion criteria. Significant cognitive impairments were observed in attention, executive function, and memory (N=78). Key findings included mixed evidence of gray matter (N=16) and white matter volume changes (N=20), cortical thickness alterations (N=9), variations in functional connectivity (N=14), electrophysiology (N=9), and blood flow (N=8). NIBS, including transcranial magnetic stimulation (N=8) and transcranial direct current stimulation (N=2), showed potential benefits for managing depression and cognitive impairments. Although cognitive rehabilitation (N=3) showed promise, it requires further investigation.

**Conclusions:**

This review highlights the complex neurologic underpinnings of PCC and the potential of NIBS and cognitive rehabilitation as interventions. Further research is essential to refine these interventions and establish evidence-based strategies for addressing long-term cognitive and psychological effects of PCC.

The COVID-19 pandemic has led to over 775 million infections worldwide, with 1 in 12 individuals (65 million) estimated to experience long-term symptoms.^1^^,^^2^ This condition, referred to as post-COVID-19 condition (PCC), post-COVID-19 syndrome, long COVID, and postacute sequelae of SARS-CoV-2 infection, lacks a universally accepted definition. Although organizations such as the World Health Organization,^3^ the National Institute for Health and Care Excellence,^4^ and the Centers for Disease Control and Prevention^5^ use different reference durations for long COVID, we adopt the term PCC for consistency and report on the definitions employed by the studies reviewed. PCC affects individuals regardless of the severity of their symptoms in the acute phase or preexisting health conditions, with an estimated prevalence of 10%-30% among nonhospitalized cases, 50%-70% in hospitalized cases, and 10%-12% among vaccinated cases.^2^ It is predominantly reported in individuals aged 36-50 years, particularly women, with many experiencing mild acute symptoms.^2^

A diverse array of neurologic complications has been documented in PCC, including symptoms such as self-reported and objective cognitive deficits,^6^ headache, anosmia, dysgeusia, dizziness, agitation, confusion, impaired consciousness, and acute stroke.^7^ Because the use of standardized assessments increases, research continues to highlight prevalent impairments in attention, executive function, and memory in PCC.^8^, ^9^, ^10^, ^11^, ^12^ These cognitive symptoms often co-occur with anxiety and depression^13^ and may persist even in the absence of significant structural abnormalities or elevated inflammation markers,^14^ further diminishing the quality of life.^15^

Neuroimaging research in PCC has yielded mixed findings, with some studies reporting a reduction in gray matter volume (GMV), cortical thickness, and/or cerebral blood flow in comparison to neurotypical controls, whereas others reported an increase in GMV in specific regions of interest, for example, the insula and the hippocampus, and white matter hyperintensities (WMH).^2^ However, the relationship between these changes and the cognitive and psychological impairment observed in individuals with PCC remains unclear. Research methodologies, such as magnetic resonance imaging (MRI), functional MRI (fMRI), positron emission tomography (PET), computerized tomography, and electroencephalography (EEG), are essential for elucidating the neural correlates of PCC.

Given the complexity and variability of PCC symptoms, various treatment approaches are being explored. Cognitive rehabilitation, a structured intervention for cognitive deficits in conditions such as traumatic brain injury and dementia,^16^ is emerging as a potential strategy for PCC. However, its effectiveness in this context remains under investigation. Similarly, noninvasive brain stimulation (NIBS) techniques, including transcranial magnetic stimulation (TMS) and transcranial direct current stimulation (tDCS), have shown promise in psychiatric and neurologic disorders^17^^,^^18^ and may hold potential for PCC symptom management.

This review aims to systematically explore and synthesize the neuroimaging and neurophysiological changes underlying PCC-related cognitive and psychological symptoms and evaluate the emerging role of NIBS and cognitive rehabilitation as therapeutic interventions.

## Methods

### Protocol registration

The review investigation adhered to the guidelines outlined in the preferred reporting items for systematic reviews and meta-analyses.^19^ The review protocol was preregistered in the International Prospective Register of Systematic Reviews (PROSPERO), assigned the registration number CRD42023475302.

### Research question

The research question under investigation, as registered in the review protocol was "What research methodologies (ie, MRI, fMRI, EEG) and treatment modalities (ie, NIBS, cognitive rehabilitation) have been used to study and address, respectively, the cognitive and psychological consequences of the post-COVID-19 condition?"

### Search strategy

Peer-reviewed research articles published before February 1, 2025, were identified using Web of Science, PubMed, and Embase. Full search terms for each database are available in the supplemental appendix S1 (available online only at http://www.archives-pmr.org/).

### Inclusion and exclusion criteria

Only peer-reviewed research articles were included. Eligible studies met the following criteria: (1) human participants aged ≥18 years, and (2) individuals with PCC. Control groups included neurotypical participants, PCC participants receiving treatment as usual or no treatment, and comparisons between different treatments (eg, NIBS vs cognitive rehabilitation). No language or publication year restrictions were applied. Excluded studies comprised case reports, prospective studies, reviews (systematic, scoping, meta-analyses), editor’s notes, and conference papers. Peer-reviewed preprints were also excluded to prevent data duplication in future updates.

### Study selection and retrieval process

In this systematic review, study selection and retrieval were facilitated using the systematic review software Rayyan.^a^ Three independent reviewers (E.P., M.L., J.T.) conducted a blinded screening of studies based on predefined inclusion and exclusion criteria. Discrepancies were resolved through discussion or consultation with the full review team (E.P., M.L., J.T., M.C., S.D.S.). Study selection followed a 2-step process: initial screening of titles/abstracts, followed by full-text evaluation. The use of Rayyan^a^ enhanced the transparency and reproducibility of the selection process.

### Risk of bias (quality) assessment

Two reviewers (M.L., E.P.) independently assessed study quality using the Newcastle-Ottawa Scale for cross-sectional and nonrandomized studies,^20^^,^^21^ the Critical Appraisal Skills Program for randomized controlled trials,^22^^,^^23^ and the Quality Appraisal Checklist for Case Series (Moga). Risk of bias scores are provided in the supplemental material (supplemental tables S1-S4, available online only at http://www.archives-pmr.org/). This rigorous assessment enhances the reliability and validity of our systematic review in the evolving field of PCC research.

### Data extraction and synthesis

Data extraction was conducted using Microsoft Excel,^b^ after a structured template adapted from a prior systematic review.^24^ The template underwent revisions and a pilot phase before finalization. Extracted data included study design, participant demographics, diagnostic tools, imaging and neurophysiological protocols (MRI/EEG/PET/computerized tomography), experimental design, intervention details, and neuroimaging, neurophysiological, neuropsychological, and psychosocial outcomes. Three reviewers (M.L., J.T., E.P.) performed data extraction, with 2 reviewers (M.C., S.D.S.) verifying accuracy. No additional information was requested from study authors. Discrepancies were resolved through team consensus (E.P., M.L., J.T., M.C., S.D.S.).

This systematic review presents a formal narrative synthesis of key findings, structured around: (1) the assessment tools, and (2) the 2 interventions—NIBS and cognitive rehabilitation—in PCC. This synthesis encompasses EEG-detected neurophysiological and structural and functional neuroimaging changes, and intervention effects. Given the limited number and heterogeneity of clinical trials, a meta-analysis was deemed infeasible. Instead, we provide a comprehensive qualitative interpretation to derive meaningful insights. Data synthesis was performed only when at least 3 eligible studies examined a specific outcome. In the case of high inconsistency or conflicting findings, deliberated on whether synthesis was appropriate.

## Results

### Study selection and retrieval process

**Fig 1:**
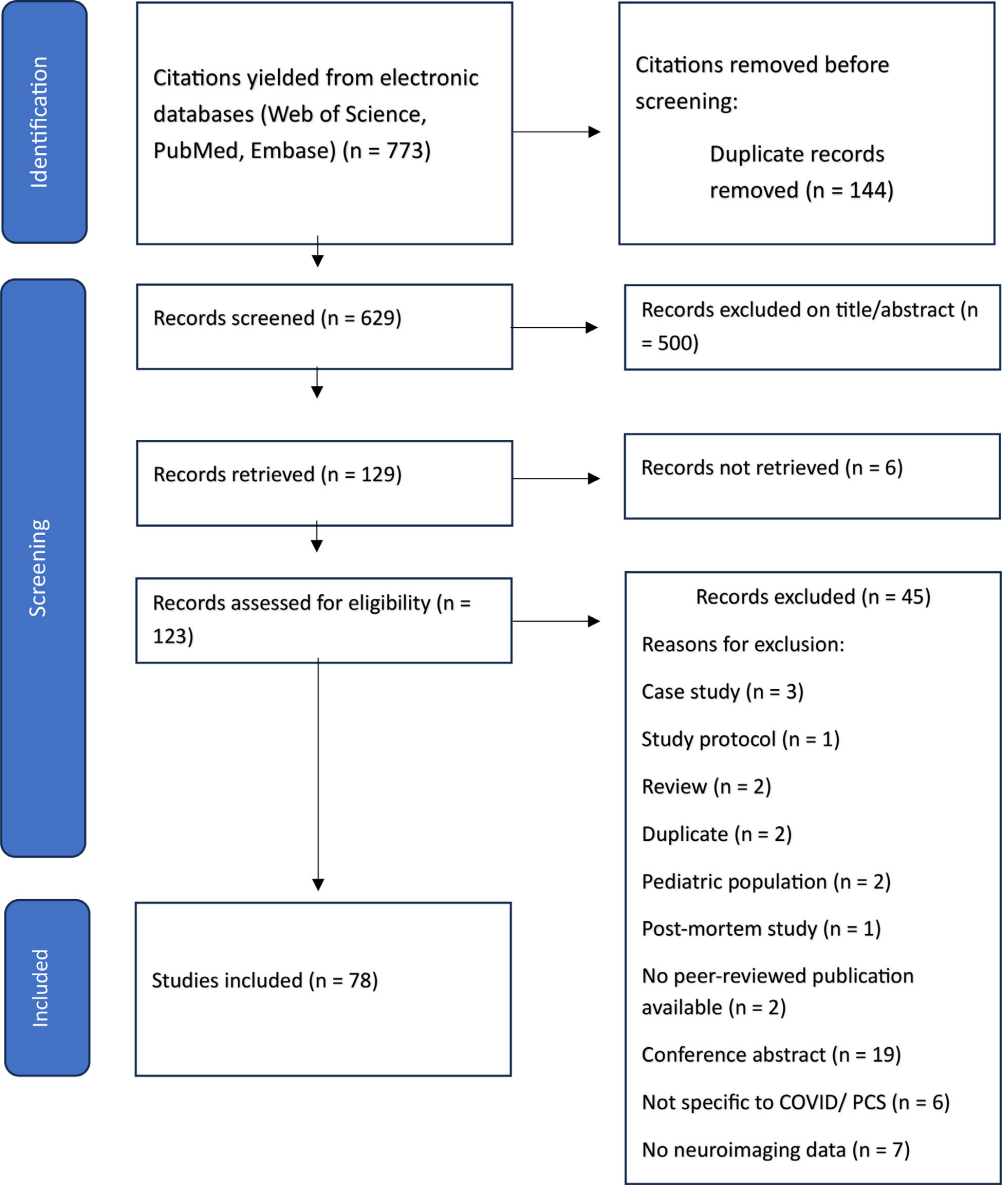
The preferred reporting items for systematic reviews and meta-analyses flow diagram of the study selection and retrieval process. Abbreviation: PCS, post-COVID-19 syndrome*.*

The electronic search identified 773 citations. After removing 144 duplicates, 629 records remained for screening. Title and abstract review led to the exclusion of 500, leaving 123 full-text articles for evaluation. Of these, 45 were excluded for various reasons (fig 1). Ultimately, 78 studies met the inclusion criteria for qualitative synthesis.

### Description of the included studies

This review includes 78 studies published between 2020 and 2025, with a total sample of over 5927 individuals (18-85y; mean, 47.69; SD, 5.79). Supplemental table S5 (available online only at http://www.archives-pmr.org/) summarizes the studies and outlines neurophysiological and psychological changes. One study collected data in April 2020,^25^ whereas the rest spanned 2021-2025.

### Study designs and sampling

Forty-nine studies^10^^,^^17^^,^^25^, ^26^, ^27^, ^28^, ^29^, ^30^, ^31^, ^32^, ^33^, ^34^, ^35^, ^36^, ^37^, ^38^, ^39^, ^40^, ^41^, ^42^, ^43^, ^44^, ^45^, ^46^, ^47^, ^48^, ^49^, ^50^, ^51^, ^52^, ^53^, ^54^, ^55^, ^56^, ^57^, ^58^, ^59^, ^60^, ^61^, ^62^, ^63^, ^64^, ^65^, ^66^, ^67^, ^68^, ^69^ used a cross-sectional study design. Six studies employed a longitudinal study design.^14^^,^^29^^,^^70^, ^71^, ^72^, ^73^ Four studies employed a double-blind design.^74^, ^75^, ^76^, ^77^ Five studies were retrospective in nature,^78^, ^79^, ^80^, ^81^, ^82^ and 3 were prospective cohort studies.^83^, ^84^, ^85^, ^86^, ^87^ Of the remaining studies, 2 were case series,^88^^,^^89^ and 2 were observational studies.^90^^,^^91^

The variation in defining PCC across studies is notable, with only a few adhering to standardized criteria. Ten studies followed the World Health Organization’s guidelines for PCC,^25^^,^^27^^,^^30^^,^^47^^,^^65^^,^^67^^,^^71^^,^^72^^,^^80^^,^^92^ whereas a single study used the Centers for Disease Control’s definition.^51^ The remaining studies defined PCC based on undefined inclusion and exclusion criteria.

### Cognitive and psychological symptoms

Of the 78 studies, 31 recorded cognitive symptoms, with 10 using a screening tool to assess cognitive impairment, either the Montreal Cognitive Assessment^25^^,^^27^^,^^30^^,^^31^^,^^34^^,^^47^^,^^50^^,^^83^ or the Mini Mental State Examination.^29^^,^^34^^,^^88^^,^^92^ In these studies, 3 showed overall significant cognitive impairment in PCC using the Montreal Cognitive Assessment cutoff score,^31^^,^^34^^,^^47^^,^^50^^,^^83^ whereas the remaining 3 studies showed significant impairment (overall or domain specific), in the group with PCC compared with the neurotypical controls.^25^^,^^27^^,^^30^^,^^50^

Thirty-four studies employed a more comprehensive cognitive assessment, with 33 reporting cognitive deficits within the groups with PCC.^14^^,^^26^^,^^28^^,^^29^^,^^33^^,^^35^^,^^36^^,^^38^, ^39^, ^40^^,^^42^^,^^44^^,^^45^^,^^49^^,^^51^, ^52^, ^53^, ^54^^,^^56^^,^^57^^,^^59^^,^^60^^,^^64^^,^^70^^,^^75^^,^^82^^,^^84^^,^^85^^,^^88^, ^89^, ^90^, ^91^, ^92^ Among these 33 studies, cognitive assessment revealed impairment in overall and specific areas of cognition, including deficits in executive functions, processing speed, attention, memory, language, and visuospatial abilities. Only 1 study reported no difference in cognitive symptoms between the group with PCC and the neurotypical controls.^17^

Similarly, 34 studies investigated the presence of affective/psychological symptoms in the groups with PCC, having recorded symptoms of anxiety,^10^^,^^14^^,^^17^^,^^27^^,^^29^^,^^34^, ^35^, ^36^, ^37^^,^^43^^,^^45^^,^^53^^,^^69^^,^^74^^,^^78^^,^^83^^,^^90^ depression,^10^^,^^14^^,^^25^^,^^27^^,^^29^^,^^34^, ^35^, ^36^, ^37^^,^^43^^,^^45^^,^^53^^,^^62^^,^^66^^,^^69^^,^^70^^,^^74^^,^^78^^,^^83^^,^^84^^,^^88^^,^^90^^,^^91^ posttraumatic stress disorder,^70^^,^^74^^,^^83^ fatigue,^29^, ^30^, ^31^^,^^34^^,^^37^^,^^39^^,^^45^^,^^53^^,^^60^^,^^65^^,^^67^^,^^74^^,^^78^^,^^81^^,^^83^^,^^84^^,^^87^^,^^88^^,^^90^^,^^91^ sleep quality dysfunction,^10^^,^^37^^,^^45^^,^^53^^,^^67^^,^^78^^,^^83^^,^^86^ and quality of life.^35^^,^^43^^,^^74^^,^^81^

### Brain structural and functional changes in PCC

Sixty-two studies investigated both structural and functional changes in the brain of individuals with PCC, using different assessment modalities including MRI for structural^10^^,^^14^^,^^17^^,^^26^, ^27^, ^28^, ^29^^,^^31^^,^^35^^,^^36^^,^^41^^,^^45^^,^^47^, ^48^, ^49^, ^50^, ^51^, ^52^, ^53^, ^54^^,^^60^^,^^64^^,^^69^, ^70^, ^71^^,^^73^^,^^82^^,^^83^^,^^85^^,^^86^^,^^87^^,^^90^ and functional^10^^,^^26^^,^^28^^,^^34^^,^^35^^,^^37^^,^^40^^,^^43^^,^^60^^,^^67^^,^^82^^,^^87^^,^^93^ changes, PET,^34^^,^^44^^,^^79^^,^^80^^,^^84^^,^^89^^,^^91^^,^^92^ and EEG.^30^^,^^32^^,^^46^^,^^48^^,^^65^, ^66^, ^67^^,^^70^^,^^72^

### Magnetic resonance imaging

Forty-four studies investigated changes in GMV, cortical thickness, mean diffusivity, fractional anisotropy, and WMH in PCC using MRI.

### Gray matter volume

Seventeen studies^10^^,^^14^^,^^26^^,^^27^^,^^35^^,^^36^^,^^45^^,^^49^^,^^50^^,^^52^^,^^55^^,^^56^^,^^63^^,^^69^^,^^70^^,^^73^^,^^82^ examined GMV changes in PCC, compared with control groups. Eleven studies reported atrophy in various brain regions, including the parahippocampal gyrus, frontal gyrus, cerebellum, occipital lobe, superior temporal lobes, posterior superior temporal gyrus, lingual gyrus, right middle occipital gyrus, thalamus, putamen, and pallidum.^10^^,^^14^^,^^26^^,^^27^^,^^36^^,^^45^^,^^49^^,^^55^^,^^56^^,^^63^^,^^82^

Gray matter (GM) atrophy in PCC was further associated with cognitive deficits, particularly in attention, processing speed, executive functions, working memory, and short-term memory.^10^^,^^14^^,^^26^^,^^27^^,^^45^^,^^55^ Atrophy in the left parahippocampal area, superior temporal gyrus, and anterior cerebellum correlated with attention and memory impairments,^10^^,^^26^ whereas thalamic, putamen, and pallidum atrophy were linked to short-term memory deficits.^27^^,^^45^ Similarly, smaller putamen volume was associated with executive dysfunction, poor sleep, and mental health issues.^55^ Additionally, verbal memory impairment correlated with total and subcortical GMV, though cognitive deficits persisted even without clear structural abnormalities.^14^ Notably, no significant associations were found between psychological symptoms and GMV in altered regions.^45^

Although overall GMV was reduced in PCC, certain regions exhibited increased volume.^26^^,^^36^^,^^69^ Specifically, greater GMV was observed in the hippocampus and thalamus^36^ and, in some cases, in the right inferior frontal gyrus, insula, orbital gyrus, temporal and middle frontal gyri, left amygdala, putamen, pallidum, postcentral and precentral gyrus, right fusiform gyrus, parahippocampal region, thalamus, caudate nucleus, substantia nigra, and ambient gyrus.^26^ Joshi et al,^69^ further reported increased GMV in the bilateral posterior cingulate, right isthmus cingulate, and combined total cingulate, though these findings did not survive multiple comparisons correction. Despite these localized increases, median whole-brain and forebrain parenchyma volumes did not differ from controls.^36^

Other studies found subtler or mixed results. Petersen et al^73^ examined olfactory bulb volume, reporting no significant differences between individuals with and without self-reported olfactory dysfunction, though lower olfactory bulb volume at baseline was linked to persistent olfactory deficits at follow-up. Ruzicka et al^52^ found mild GMV reductions in some but not all individuals with PCC. Finally, 3 studies found no significant differences in GMV between PCC and control groups.^35^^,^^50^^,^^70^

### Cortical thickness

Nine studies^28^^,^^47^^,^^54^^,^^64^^,^^69^^,^^85^^,^^86^ investigated cortical thickness changes in PCC. Several reported cortical thinning^28^^,^^47^^,^^54^^,^^85^^,^^86^ in bilateral mean cortical thickness, lower subcortical GM, and lower right olfactory bulb volume,^85^ as well as region-specific reductions in the lateral orbitofrontal cortex, parahippocampal region, caudal middle frontal cortex, medial orbitofrontal cortex, lingual gyrus, pericalcarine cortex, and lateral occipital regions.^28^^,^^47^^,^^54^^,^^64^^,^^85^^,^^86^

Thinning in the lateral orbitofrontal cortex correlated with olfactory deficits,^54^^,^^85^ and anosmia was linked to a bilateral cortical thickness reduction.^85^ Lower cortical thickness in the left parahippocampal and right caudal middle frontal regions correlated with memory impairment.^64^ Progressive cortical thinning was observed across individuals, from COVID-negative controls to COVID survivors and patients with PCC with cognitive impairment.^47^

In contrast, some regions showed increased cortical thickness in PCC. Joshi et al^69^ reported bilateral thickening in the caudal anterior cingulate, right posterior cingulate, right isthmus cingulate, and right rostral middle frontal gyrus; however, these findings did not survive multiple comparisons correction. Besteher et al^47^ found a continuum of cortical thickening in patients with PCC with more pronounced cognitive impairment, affecting prefrontal, temporal, parahippocampal, insular, and parietal areas.

Despite these findings, Muccioli et al^28^ did not detect any differences in cortical thickness while also noting symmetry in cortical thickness between the right and left hemispheres.

### Magnetic resonance spectroscopy

One study^41^ investigated deep brain GM changes and specifically, metabonomic mapping, with individuals with PCC brain fog exhibiting changes in their Glx and Lac concentration compared with neurotypical controls.

### Arterial spin labeling

One study^31^ conducted arterial spin labeling revealing that the global mean of cerebral blood flow in GM was lower in the group with PCC compared with the neurotypical controls. Mean cerebral blood flow was significantly lower in the bilateral frontal, right parietal, and bilateral temporal lobes in the group with PCC. Additionally, they reported no significant differences in the left parietal and bilateral occipital lobes and cerebellum, as well as no associations between cognitive impairments, assessed with the Montreal Cognitive Assessment, and cerebral blood flow within the group with PCC.^31^

### Diffusion tensor imaging

Ten studies^10^^,^^17^^,^^45^^,^^49^^,^^57^, ^58^, ^59^, ^60^^,^^71^^,^^87^ reported reductions in fractional anisotropy and increases in mean, radial, and axial diffusivity in patients with PCC compared with neurotypical controls. These changes predominantly affected the right hemisphere but were also observed bilaterally across frontal, temporal, parietal, occipital, and subcortical regions.^10^^,^^57^ Although weakly associated with cognitive dysfunction, these alterations were more pronounced in individuals who had been hospitalized during acute infection.^10^ Similarly, Liang et al^17^ found increased fractional anisotropy and decreased diffusivity across multiple white matter (WM) tracts, with women displaying significantly greater left amygdala mean diffusivity than controls. Changes in fractional anisotropy within the thalamus correlated with fatigue-related symptoms, including physical fatigue, impairment in daily activities, and daytime sleepiness.^45^

Further analyses confirmed widespread disruptions in WM integrity. Serrano del Pueblo et al^49^ observed lower fractional anisotropy and higher radial diffusivity in patients with PCC, with these abnormalities linked to episodic memory deficits, impaired cognitive function, attentional difficulties, and reduced verbal fluency. Similarly, in Ref. [58] significantly lower mean diffusivity in patients with PCC was identified, particularly in the internal capsule, corona radiata, corpus callosum, superior fronto-occipital fasciculus, and posterior thalamic radiation. However, the effect sizes of these differences were small, and fluid cognition composite scores remained comparable to controls. Churchill et al^58^ also reported WM alterations, including reduced mean and axial diffusivity and increased mean kurtosis and neurite dispersion in deep WM. These changes were further associated with greater negative affect, which correlated with increased mean kurtosis and reduced free water in WM. Meanwhile, NeuroPASC participants exhibited larger cerebral WM volume, primarily in the prefrontal and anterior temporal regions, as well as increased mean kurtosis, suggesting ongoing neuroinflammation.^59^

A longitudinal study^71^ indicated that WM abnormalities persisted for up to 2 years postinfection, despite large-scale recovery trends. Initially, higher levels of inflammation correlated with greater WM abnormalities and lower cognitive function. Over time, small-scale regional deterioration continued, and the extent of WM damage remained significantly linked to cognitive deficits.^71^ Dacosta-Aguayo et al^87^ demonstrated a significant negative correlation between radial diffusivity and memory performance, though fractional anisotropy and axial diffusivity showed no associations. Similarly, Diez-Cirarda et al^60^ identified WM diffusivity correlates for fatigue and subjective cognitive complaints, particularly in the forceps minor, anterior corona radiata, and anterior cingulum, further reinforcing the link between structural abnormalities and persistent symptoms in PCC.

In summary, these 10 studies suggest that PCC is characterized by widespread WM disruptions, including reduced fractional anisotropy and increased diffusivity. While consistently observed across multiple brain regions, their clinical relevance varies, with some studies linking them to cognitive deficits, fatigue, and emotional disturbances. Findings from both cross-sectional and longitudinal studies indicate that these alterations may persist long after infection, potentially contributing to chronic symptoms. However, variability in effect sizes and cognitive associations underscores the need for further research to clarify their effect on symptom severity and recovery.

### Diffusion-weighted imaging

Two studies investigated brain hallmarks of PCC with diffusion-weighted imaging. Saleh and Shaban^90^ reported that the most prevalent MRI findings in individuals with PCC included cerebrovascular stroke, with infarction observed in 37.14% of cases, hematoma in 20%, vasculitis in 15.7%, demyelinating diseases in 14.28% (comprising acute disseminated encephalomyelitis at 7.1% and posterior reversible encephalopathyat 7.1%), gyral edema in 5.7%, and common WM micro-hemorrhages in 2%. MRI failed to detect abnormalities in 2 cases despite 1 patient experiencing severe headaches and the other exhibiting prolonged fatigue. The correlation between neurologic changes, measured with diffusion-weighted imaging, and respiratory symptoms in PCC was notably weak.^90^ Conversely, none of the participants with PCC exhibited diffusion-weighted imaging lesions.^48^

### Susceptibility-weighted imaging

One study employed susceptibility-weighted imaging to detect cerebral microbleeds in individuals recovering from COVID-19.^83^ It revealed that 61% of the participants who had been admitted to the intensive care unit during infection exhibited microbleeds, a significantly higher prevalence compared to nonintensive care unit participants. Moreover, intensive care unit participants had a greater number of microbleeds, particularly located in the corpus callosum, a region known to be vulnerable to hypoxic and microvascular injury. There were no significant differences in other MRI findings between the 2 groups, and no group-level differences in cognitive performance were observed. Additionally, there was no significant relationship between the number or distribution of microbleeds and cognitive test performance.^83^

### White matter hyperintensities

WMH in PCC and neurotypical controls has been investigated reporting structural abnormalities.^29^^,^^50^^,^^51^^,^^53^^,^^70^^,^^82^^,^^87^ Increased WMH volumes were observed in patients with PCC, particularly in the right frontal and parieto-occipital regions, with greater WMH burden in the left parieto-occipital region correlating with lower performance in immediate and delayed recall tasks.^70^ In addition, WMH presence in the right hemisphere—including the superior frontal region, postcentral region, cingulum, corticospinal tract, inferior longitudinal fasciculus, internal capsule, and posterior segment of the arcuate fasciculus—was associated with poorer performance in action speed, language, and executive function.^29^ However, although total WMH burden significantly correlated with cognitive function, the presence of microbleeds did not.^29^

Mild microangiopathic WM changes, consistent with Fazekas I (ie, punctate periventricular or deep WMH), were detected in patients with PCC,^50^ with some cases exhibiting small gliotic lesions, postischemic cortical defects, or slight T2 signal elevations in the globus pallidum. However, no clear signs of atrophy or inflammation were identified. Similarly, Kurakh et al^51^ categorized WMH severity using the Fazekas scale, noting that 13 patients exhibited mild periventricular changes (Fazekas I), whereas 5 showed moderate lesions and widened lateral ventricles (Fazekas II). Only 3 patients had severe degenerative WM changes (Fazekas III), characterized by multiple focal lesions with confluence and widened subarachnoid spaces.

Hu et al^53^ further classified WMH patterns in patients with PCC, identifying 5 distinct types, including periventricular WMHs (Fazekas 0-II), deep subcortical WMHs, isolated ovoid WMHs, bilateral medial temporal WMHs, and pontine WMHs. Notably, individuals with ovoid WMHs exhibited relatively preserved memory compared with those with bitemporal WMHs or those without COVID-associated WMH patterns, though these differences were subtle. Despite these variations, MRI findings alone were insufficient to predict cognitive and noncognitive impairments.

Dacosta-Aguayo et al^87^ linked WM abnormalities to cognitive deficits, with over half of PCC participants displaying impairments in attention (55%) and executive function (59%), while 40% exhibited memory dysfunction. No significant differences were observed in global MRI metrics, including brain parenchymal fraction, GM fraction, and cortical thickness, between patients with PCC and controls. However, they reported an increased prevalence of WMHs in subcortical WM, the centrum semiovale, and the internal capsule, with a statistically significant rise in juxtacortical WMHs in mild COVID-19 cases compared with controls.

Summarizing these findings, the studies indicate an increased WMH burden in PCC, particularly in frontal and parieto-occipital regions, with strong associations to cognitive impairments in recall, processing speed, language, and executive function. However, microbleeds showed no clear link to cognitive dysfunction. Although some studies reported mild microangiopathic changes, others identified advanced WM degeneration in select cases. These findings suggest WMH as a potential biomarker for cognitive deficits in PCC, though variability in severity and distribution warrants further investigation into their clinical relevance and long-term effect.

### Functional magnetic resonance imaging

Thirteen of the studies retrieved for this systematic review investigated functional changes in PCC with fMRI.^10^^,^^26^^,^^28^^,^^34^^,^^35^^,^^37^^,^^40^^,^^43^^,^^60^^,^^67^^,^^82^^,^^87^^,^^93^ Reduced functional connectivity (FC) was observed between the left and right parahippocampal gyrus, as well as between the left cerebellar III and bilateral frontal superior orbital cortices.^10^ These alterations were more pronounced in hospitalized individuals and were significantly associated with poorer memory recall performance.^10^ Similarly, hippocampal atrophy in patients with PCC was accompanied by microstructural changes, hypoperfusion, and disrupted FC, particularly in regions linked to memory and attention.^26^

Disruptions were also observed in the olfactory and thalamic networks. The global modularity coefficient of the olfactory subnetwork was significantly reduced in PCC, with increased local connectivity in the right thalamus, particularly in its connections to the right posterior hippocampus and right insula.^28^ In individuals with anosognosia, hypoconnectivity was recorded between the left lateral prefrontal cortex in the default mode network and the left somatosensory motor cortex, bilateral frontal eye field, and dorsal attention network, as well as between the somatosensory motor network and cerebellar regions.^35^

Connectivity changes also correlated with cognitive and emotional processing. In cortico-subcortical-cerebellar networks, FC patterns associated with multimodal emotion recognition varied depending on PCC severity, with positive correlations in mild cases, negative correlations in moderate cases, and no significant associations in severe cases.^37^ Widespread reductions in FC were also observed across subcortical, medial temporal, frontal, and parietal regions, with the most affected areas including the thalamus, parahippocampal gyri, amygdala, basal ganglia, and superior temporal gyri. Altered connectivity was particularly pronounced in intratemporal, intrasubcortical, and frontal-subcortical regions, with symptom severity correlating with the degree of FC disruption.^40^

Additional fMRI analyses revealed universal hypometabolism in the parietal, temporal, frontal, and occipital lobes, as well as the thalamus, aligning with PET scan findings^34^ (see next section). In contrast, task-based fMRI using the N-back paradigm demonstrated greater activation in the superior frontal gyrus and reduced deactivation of default mode-related regions in patients with PCC, indicative of altered neural engagement during cognitive tasks.^43^

FC alterations were also linked to fatigue and sleep disturbances. Fatigue-related connectivity changes were observed primarily in frontal, temporal, and cerebellar areas, with distinct patterns differentiating mental and physical fatigue.^60^ FC between the middle temporal pole and ventral tegmental area, as well as between the cerebellar peduncle and cerebellum, predicted sleep disturbances at follow-up, whereas connectivity between the dorsal raphe nucleus and occipital, parietal, and temporal regions was associated with daytime dysfunction.^67^

Memory-related changes were also evident. Increased activation in the frontoparietal network, including the superior and middle temporal gyri and posterior cingulate cortex, was negatively correlated with memory performance.^87^ Niemczak et al^93^ found that patients with PCC exhibited greater frontal activation during cognitively demanding tasks, particularly in the 2-back working memory paradigm. Similarly, González-Rosa et al^82^ reported hypoconnectivity in both the default mode network and dorsal attention network in mild COVID-19 cases, particularly in the left precuneus, cuneus, and right angular gyrus compared with controls.

In summary, fMRI studies reveal widespread FC disruptions in PCC, particularly in networks governing memory, attention, and emotion. Reduced connectivity in the hippocampus, thalamus, and parahippocampal regions correlates with cognitive deficits, whereas alterations in olfactory and thalamic networks suggest sensory processing abnormalities. Task-based fMRI findings indicate compensatory neural engagement, possibly reflecting cognitive inefficiencies. Connectivity changes linked to fatigue and sleep disturbances suggest distinct neural signatures for these symptoms. These findings highlight the complexity of PCC-related brain dysfunction and the need for further research on reversibility and symptom persistence.

### Positron emission tomography

Eight studies employed PET to examine changes in brain activity in PCC participants, ^34^^,^^44^^,^^79^^,^^80^^,^^84^^,^^89^^,^^91^^,^^92^ with 1 study revealing no significant changes in brain activity between PCC participants and controls.^84^ The remaining 7 studies reported significant activity changes in various brain regions. The study^44^ showed significantly higher cerebellar metabolism. Evidence of significant hypometabolic reduction was reported for multiple areas including the frontal, parietal, temporal, and occipital lobes and the thalamus;^34^ the locus coeruleus;^89^ and the bilateral rectal/orbital gyrus, including the olfactory gyrus, the right temporal lobe, including the amygdala and the hippocampus, extending to the right thalamus, the bilateral pons/medulla brainstem, and the bilateral cerebellum.^79^ Findings from Martini et al^92^ also showed significant hypometabolic reduction in the left middle−superior and orbital frontal gyri and parietal regions, which appeared to dissolve over time as reassessed at 5 months leaving a slight residual hypometabolic cluster in the right superior and middle frontal cortex and medial frontal cortex and at 7-9 months leaving no residual damage.^92^ In Ferrucci et al,^80^ 3 of 7 participants with PCC showed various brain hypometabolism patterns, including in the unilateral left temporal mesial area, the pontine involvement, and the bilateral prefrontal and parietal areas. The patient with the most widespread glucose hypometabolism also presented with significant Aβ deposition in the superior and middle frontal cortex and the posterior cingulate cortex, extending mildly in the rostral and caudal anterior cingulate areas.^80^ Finally, hypoperfusion in the bilateral occipital and frontal lobes was observed on single-photon emission computed tomography in the group with PCC.^91^

### Electroencephalography

Nine studies consistently reported altered electrophysiological activity in PCC, with differences in spectral power, event-related potentials, and connectivity patterns compared with neurotypical controls.^30^^,^^32^^,^^46^^,^^48^^,^^65^, ^66^, ^67^^,^^70^^,^^72^ Participants with PCC exhibited lower individual α frequency and greater current source densities in the δ frequency band, particularly in bilateral frontal and central-temporal regions.^70^ Reduced δ activity, alongside slight reductions in theta activity, was also reported.^30^ Additionally, event-related potential alterations were noted, with differences in P300 latency and amplitude,^32^ as well as event-related potential signals in patients with PCC with and without brain fog compared with neurotypical controls.^46^ Abnormal EEG signals were detected in 65% of PCC participants, with 69% showing decreased activity and 31% presenting epileptic discharges, predominantly in the frontal regions.^46^

Altered β and γ oscillatory activity was observed in frontoparietal and frontocentral regions. Gangemi et al^72^ reported significant differences in P300 potentials and β band rhythms in individuals with cognitive fog, which persisted up to 8 months post-COVID-19. Similarly, others^65^ found a significant reduction in β- and γ-range activity in frontoparietal areas after motor cortex (M1) and supplementary motor area stimulation. Cluster-based analyses revealed reductions in β-transient response spectral perturbation in frontocentral electrodes in the PCC group compared with controls, with these reductions correlating with higher fatigue scores.

Resting-state EEG findings further demonstrated reduced posterior α power in PCC participants, particularly in those experiencing fatigue.^67^ However, Babiloni et al^66^ found no significant differences in the amount of artifact-free resting-state EEG epochs between post-COVID and control groups.

Summarizing these findings, the EEG studies reveal widespread electrophysiological disruptions in PCC, including altered spectral power, event-related potentials, and cortical excitability. Reduced α and δ activity, along with P300 abnormalities and increased epileptic discharges, suggest impaired neural processing linked to cognitive dysfunction and fatigue. Changes in β and γ oscillations further indicate disruptions in frontoparietal networks. Although these findings highlight the neurophysiological effect of PCC, their variability across studies underscores the need for further research to establish reliable biomarkers and track their progression over time.

### The effectiveness of treatment modalities in cognitive and psychological difficulties in PCC

#### Noninvasive brain stimulation

Ten studies implemented NIBS protocols to improve cognitive and/or psychological symptoms in participants with PCC, with 8 reporting the use of TMS,^25^^,^^33^^,^^38^^,^^39^^,^^75^^,^^78^^,^^88^^,^^91^ and 2 employing tDCS.^76^^,^^77^

#### Transcranial magnetic stimulation

In the first study to be described here,^25^ recorded motor-evoked potentials (MEPs) from the first dorsal interosseous muscle after single-pulse TMS revealed smaller baseline compound muscle action potential amplitudes in patients with PCC. Although MEP amplitudes significantly decreased posttask in controls, they remained unchanged in PCC. Additionally, the silent period was extended in controls but shortened in PCC, suggesting altered motor cortex physiology.

Similarly, Versace et al^33^ detected reduced inhibition in the primary motor cortex, indicated by impaired γ-aminobutyric acid (GABAa)-mediated short-interval intracortical inhibition and GABAb-mediated long-interval intracortical inhibition, while intracortical facilitation remained unaffected. Another study^38^ further reported reduced long-interval intracortical inhibition and intracortical facilitation in patients with PCC undergoing paired-pulse TMS and short-latency afferent inhibition, highlighting disruptions in GABABergic and glutamatergic pathways.

Patients with PCC exhibited higher perceived exertion and smaller MEPs during a pinching task, despite no significant differences in maximum force, resting motor threshold, or intracortical facilitation compared with controls.^39^ However, reduced long-interval intracortical inhibition and sensory afferent inhibition suggested compromised motor cortex excitability and potential cognitive impairments.

Individuals with PCC diagnosed with anxiety and/or depression received a 20-session intervention combining intermittent θ burst stimulation for the dorsolateral prefrontal cortex (DLPFC) and low-frequency repetitive TMS (rTMS) for the right lateral orbitofrontal cortex.^88^ Men showed greater reductions in depressive symptoms, while overall improvements were noted in subjective and objective depressive symptoms, mild fatigue, and brain fog. Similarly, rTMS targeted the DLPFC with protocols tailored to individual symptoms, resulting in significant improvements in depression, insomnia, anxiety, and other neuropsychiatric symptoms.^78^

Double-blind, placebo-controlled trial examined the effects of an 8-week palmitoylethanolamide and luteolin intervention alongside TMS.^75^ The palmitoylethanolamide and luteolin group exhibited increased GABAergic activity in M1, reflected in enhanced inhibition of conditioned MEPs and long-term potentiation-like cortical plasticity, though no significant changes were observed in resting motor threshold or cognitive test outcomes.

Finally, another study^91^ implemented an rTMS protocol targeting 2 midline sites on the forehead and occipital region over 10 sessions (24,000 stimuli total). Participants demonstrated improvements across all Wechsler Adult Intelligence Scale–Fourth Edition subtests, increased full-scale intelligence quotient, and reduced apathy and fatigue scores.

#### Transcranial direct current stimulation

Two studies investigated the effects of tDCS on PCC-related symptoms, both targeting the left DLPFC using a neuronavigation system for precision.^76^^,^^77^

In one study,^76^ 47 participants with PCC were randomized into active (n=23) and sham (n=24) tDCS groups, undergoing 8 stimulation sessions over 2 weeks. The active tDCS group demonstrated a significant reduction in physical fatigue immediately posttreatment and at 1-month follow-up, as well as improvements in depressive symptoms. However, no significant effects were observed for cognitive fatigue or overall quality of life.

Similarly, Klírová et al^77^ conducted a 4-week randomized controlled trial and found a general reduction in fatigue severity over time across both groups, though within-group improvements were significant only in the sham condition. No significant differences between active and sham tDCS groups were observed for cognitive, physical, and psychosocial fatigue domains, post-COVID symptom burden (Adapted Postacute Sequelae of SARS-CoV-2 scores), or anxiety and depressive symptoms (General Anxiety Disorder 7-item Scale, Patient Health Questionnaire 9). Cognitive function, measured through attention, working memory, and psychomotor tests, showed no differences between groups. Mild to moderate side effects, such as tingling or burning sensations, were reported in both groups during the initial treatment phase but subsided over time. No persistent adverse effects were noted at follow-up.

### Cognitive training combined with NIBS

Three studies were identified that conducted cognitive training.^68^^,^^74^^,^^94^ Santana et al^74^ investigated the effects of cognitive training combined with tDCS in PCC. All participants underwent individually tailored rehabilitation for fatigue, with half receiving active tDCS and the other half undergoing sham tDCS. The intervention lasted 5 weeks, with 2 sessions per week. The active tDCS group showed significantly greater improvements in cognitive and psychosocial fatigue but not in physical fatigue. Additionally, anxiety symptoms were reduced, and quality of life improved in the active tDCS group compared with the sham group, while no significant differences were observed in pain measures.

Nagy et al^68^ examined neural dynamics before and after cognitive training using resting-state EEG. Pretraining assessments revealed no differences between post-COVID men and women. However, posttraining analyses showed that cognitive training selectively enhanced neural processing in women, increasing neural signal complexity and oscillatory power. Post-COVID women initially exhibited increased finer timescale entropy (1-30ms) and higher frequency band power (11-40 Hz) compared with healthy controls, but these differences disappeared after cognitive training. No such effects were observed in men.

Deodato et al^94^ evaluated the effects of dual-task augmented reality rehabilitation on cognition and fatigue in patients with long COVID. Ten individuals with cognitive impairment and fatigue underwent 21-hour training sessions and were compared with a control group of 10 patients with long COVID who did not receive rehabilitation. Cognitive performance improved in the training group, with significantly faster Trail Making Test Part B completion times and higher Frontal Assessment Battery scores. No significant differences between groups were found for fatigue severity, cardiovascular endurance, or muscular fatigue.

In summary, NIBS appears to hold promise for alleviating cognitive and psychological symptoms in individuals with PCC. rTMS targeting the DLPFC or motor areas showed improvements in cognitive function, depressive symptoms, fatigue, and apathy, though effects varied depending on stimulation site and protocol. tDCS yielded more mixed results, with some studies reporting reduced fatigue and improved mood, whereas others observed minimal differences from sham stimulation. Combining cognitive training with NIBS, especially in targeted subgroups (eg, older women), showed potential for enhancing neural efficiency and cognitive performance. Although findings are encouraging, further large-scale, controlled trials are needed to optimize protocols and clarify treatment efficacy.

## Discussion

The synthesis of findings from this systematic review illuminates the complex interplay of structural, functional, and neurophysiological changes in the brain associated with PCC, alongside emerging evidence on NIBS and cognitive training as potential interventions. Here, we discuss the implications of these findings, propose future research directions, and highlight the need for targeted interventions.

The discrepancy in defining PCC across research studies presents a major challenge because the lack of uniformity in inclusion criteria affects result interpretation. Although a minority of studies adhere to established guidelines by the World Health Organization^3^ or the Centers for Disease Control and Prevention,^5^ most rely on unique self-defined inclusion criteria. This inconsistency hinders meta-analyses, limits reproducibility, and complicates patient stratification for clinical interventions. Standardizing definitions is essential for improving research cohesion, enabling accurate epidemiologic assessments, and guiding precision-based interventions.

A recapitulation of global trends indicates a continuum of cortical thickness alterations associated with COVID-19, with pronounced changes in individuals experiencing cognitive impairments.^14^ This observation, coupled with variations in GMV and cerebral blood flow, hints at the heterogeneity of PCC’s neurologic effect. Summarizing these trends aids in digesting the multitude of findings, suggesting that PCC may indeed lead to distinct clinical subtypes, each characterized by unique patterns of brain alterations and symptomatic presentations.^6^

The observed larger GMV in certain brain regions among patients with PCC may indicate underlying recovery mechanisms or inflammatory activity.^11^ This duality of interpretation reflects the complexity of PCC, where increased GMV could represent compensatory neuroplasticity or ongoing pathologic processes. The findings advocate for a nuanced understanding of PCC, where both detrimental and potentially adaptive neurologic changes coexist, delineating subtypes of the condition with implications for tailored interventions.^8^^,^^9^

The evidence of cortical thickness alterations forming a continuum, particularly notable in individuals with cognitive deficits, highlights the profound effect of COVID-19 on the brain’s structural integrity.^10^ The variability in the alterations associated with cognitive impairments underscores the need for targeted therapeutic strategies that address both the neurobiological and functional aspects of the disease. The specificity of these changes calls for precision medicine approaches in the management of PCC, leveraging detailed neuroimaging findings to guide intervention choices.^7^

TMS shows significant benefits for depression and anxiety in PCC, yet the interaction with fatigue severity presents a crucial consideration for clinical practice.^13^ The diminished efficacy of TMS in patients with severe fatigue suggests that intervention planning should account for the multifaceted symptomatology of PCC, where fatigue emerges as a pivotal factor influencing therapeutic outcomes. This finding prompts further investigation into the mechanisms by which fatigue modulates response to TMS, potentially guiding the development of adjunct or alternative therapies to optimize mental health outcomes in this population.^15^

The absence of studies combining cognitive training with NIBS in PCC underscores a significant gap in the current research landscape. This review suggests the potential for an integrated approach that combines cognitive training with NIBS, such as TMS or tDCS, to enhance cognitive rehabilitation outcomes. This proposition is based on the premise that combining modalities could synergize to target both the neurophysiological underpinnings and the cognitive manifestations of PCC, potentially offering a more comprehensive intervention strategy.^2^

### Study limitations

This systematic review possesses notable strengths. It comprehensively integrates structural and functional neuroimaging, neurophysiological assessments, and intervention studies, providing a multidimensional perspective on PCC-related cognitive and psychological impairments. The rigorous adherence to preferred reporting items for systematic reviews and meta-analyses guidelines and the use of validated quality assessment tools enhance the robustness and reliability of the findings. The inclusion of advanced imaging modalities (MRI, fMRI, PET, EEG), alongside emerging interventions such as NIBS and cognitive rehabilitation, highlights innovative strategies and broadens the scope of therapeutic exploration. Moreover, this review integrates data from studies conducted across various geographic and temporal contexts, enhancing the global relevance and generalizability of its findings. Despite these strengths, this review is not without limitations. The heterogeneity in diagnostic criteria, participant characteristics, and assessment tools limits the capacity for quantitative synthesis and introduces potential biases. The paucity of longitudinal studies further restricts insights into the trajectory of PCC and its response to therapeutic interventions over time. Furthermore, the small number of high-quality randomized controlled trials on NIBS and cognitive training limits definitive conclusions on treatment efficacy. Lastly, the exclusion of non-peer-reviewed preprints and non-English studies may introduce publication bias, potentially omitting relevant findings.

## Conclusions

This systematic review underscores the importance of a multidimensional approach to understanding the neurologic underpinnings of PCC and treating the neuropsychological symptoms of PCC, highlighting the potential for novel intervention combinations, the need for personalized intervention strategies, and the imperative for future research to unravel the complex neurologic and psychological landscape of this condition. Because we advance our understanding of PCC’s multifarious effects, the insights garnered from this review pave the way for more effective, tailored interventions that address the unique needs of affected individuals, fostering improved outcomes and quality of life.^12^

## Suppliers

a. Rayyan software for systematic reviews; Rayyan.
b. Excel, version 16.0; Microsoft.

## Appendix Supplementary materials

Image, application 1

Image, application 2

## Footnotes

Supplementary material associated with this article can be found, in the online version, at doi:10.1016/j.arrct.2025.100461.
