# Supplementary material for: Cognitive and Psychological Symptoms in Post-COVID-19 Condition: A Systematic Review of Structural and Functional Neuroimaging, Neurophysiology, and Intervention Studies
Source: Arch Rehabil Res Clin Transl. 2025 May 9;7(3):100461. doi: 10.1016/j.arrct.2025.100461 (PMC12447218; doi:10.1016/j.arrct.2025.100461)
Supplement: Supplementary file 1 [file mmc1.docx]

Supplemental Table S5. A summary of the studies and outlines neurophysiological and psychological changes.

| **Study first author** | **Study design** | **Sample** | **Description of intervention/ protocol** | **Neuroimaging/ neurostimulation methods** | **Main findings** |
| --- | --- | --- | --- | --- | --- |
| Ajcevic  (2023) | Cross-sectional | N = 46  (n = 24 PCC patients  n = 22 healthy controls) | N/A | MRI | Significant hypoperfusion in frontal, temporal, and parietal cortex of PCC patients with cognitive impairment, approximately 2-10 months after the onset of symptoms. |
| Alkhormani  (2024) | Analytical cross-sectional | N = 428  (n = 327 fully recovered within 14 days,  n = 71 prolonged symptoms resolving within 28 days,  n = 29 persistent symptoms beyond 28 days) | N/A | N/A | Symptom differences: Acute phase: Headache, loss of smell/taste, and fatigue were significantly more frequent in those with prolonged/persistent symptoms. Post-COVID phase: Loss of smell/taste and shortness of breath were more common in the persistent symptoms group. Gender susceptibility: Females were more prone to long COVID. Mental health impact: Higher PHQ-9 scores in those with prolonged/persistent symptoms, indicating a significant association with depression. |
| Babiloni  (2024) | Case-control | N = 51  (n = 36 PCC patients,  n = 15 healthy controls) | N/A | EEG | No significant cognitive or psychiatric impairments in over 90% of PCC patients. 75% of PCC patients reported at least two fatigue symptoms. Reduced posterior alpha activity in PCC patients, especially in those with higher fatigue levels. |
| Baker  (2024) | Cross-sectional | N = 37 PCC patients with fatigue | N/A | MRI | Reduced activity in specific brain circuits. Evidence of abnormal autonomic function. Myopathic alterations detected. Cluster analysis suggested post-COVID fatigue is a single entity with individual variation, rather than multiple distinct syndromes. No evidence of dysregulation in sensory feedback circuits or descending neuromodulatory control. |
| Besteher (2022) | Cross-sectional | N = 50  (n = 30 PCC patients  n = 20 healthy controls) | N/A | MRI | Cognitive and psychological symptoms not associated with gray matter volume. Increased gray matter volume in frontal areas and some limbic structures, in PCC. Could reflect recovery mechanisms or inflammatory activity. |
| Besteher (2023) | Cross-sectional | N = 120  (n = 61 PCC patients,  n = 30 never infected controls,  n = 29 healthy COVID survivors) | Twenty-six PCC patients presented with cognitive impairment and 35 without. | MRI | Never-infected controls presented with the highest cortical thickness. PCC patients with clinically significant cognitive impairment showed the lowest cortical thickness. However, cortical thickness was not associated with cognitive performance between groups. |
| Besteher  (2024) | Cross-sectional | N = 120  (n = 30 healthy never-infected controls,  n = 29 healthy COVID-19 survivors,  n = 26 PCC patients with cognitive impairment (MoCA < 26),  n = 35 PCC patients without cognitive impairment (MoCA ≥ 26)) | N/A | MRI | Cortical thickness progressively increased from healthy controls to COVID-19 survivors, long-COVID patients without cognitive impairment, and was most pronounced in long-COVID patients with cognitive deficits. Affected brain regions included the prefrontal and temporal gyri, insula, posterior cingulate, parahippocampal gyrus, and parietal areas. Distinct immune profiles were identified in long-COVID patients, particularly those with cognitive impairment, showing elevated IL-10, IFNγ, and sTREM2 levels. |
| Bowen  (2023) | Cross-sectional | N = 14 PCC patients | Half of the patients received transcranial photomodulation, the rest received whole-body photomodulation (12 sessions). EEG was performed after the intervention | EEG & transcranial/ whole body photomodulation | Significant reduction in P300 reaction time, after transcranial photomodulation, compared to whole-body. Cognitive performance improved in both groups. |
| Casula  (2024) | Case-control study | N = 32  (n = 16 PCC patients with persistent fatigue, n = 12 healthy controls) | Participants underwent TMS-EEG to assess neural oscillatory activity in the left primary motor cortex (l-M1) and supplementary motor area (SMA) | EEG | Post-COVID patients showed reduced beta oscillatory activity in both l-M1 and SMA compared to controls. A significant inverse correlation was found between SMA beta activity and fatigue severity (higher fatigue = lower beta oscillations). |
| Cataldo  (2024) | Cross-sectional | N = 137  (n = 109 PCC patients,  n = 28 healthy controls) | N/A | MRI | Long COVID patients reported persistent cognitive symptoms, including memory issues and brain fog, along with higher fatigue and lower quality of life. Objective cognitive tests did not show significant differences between groups, except for slower performance on the Trail Making Test-A (TMT-A). MRI findings revealed reduced volume in the cerebellum, lingual gyrus, and inferior parietal regions, along with decreased cortical thickness in the postcentral gyri and precuneus. |
| Cecchetti (2022) | Longitudinal (10 months) | N = 85  (n = 36 healthy controls  n = 49 PCC patients) | N/A | EEG & MRI | At baseline, better cognitive performance was associated with better connectivity between left parieto-occipital regions and right frontal regions at delta band (PCC). PCC patients also showed greater white matter hyperintensities volume in frontal regions, that correlated with cardiovascular risk factors. At follow-up, 36% of PCC patients showed impairment in at least one cognitive domain. |
| Chang (2023) | Randomised controlled open-label trial (pilot) | N = 24 PCC patients with olfactory dysfunction | Ten participants received olfactory training 3 times per day combined with oral VitA (combined treatment), 9 patients received standard care (olfactory training) and 5 patients received clinical observation (controls), for 4 weeks | rs-fMRI | Olfactory function was significantly improved for patients in the combined treatment condition, compared to controls. Increased functional connectivity in the combined treatment group, compared to standard care and controls. |
| Churchill (2023) | Cross-sectional | N =66  (n = 51 had tested positive for COVID-19 5 months prior,  n = 15 controls with flu-like symptoms but negative for COVID-19 5 months prior) | N/A | MRI & fMRI | Cognitive and memory symptoms most consistently reported in the COVID group. Decreased functional connectivity in subcortical and medial temporal regions for the COVID group compared to controls. |
| Churchill (2024) | Cross-sectional, observational | N=68  (n = 54 PCC patients  n = 14 healthy controls) | N/A | MRI | PACS patients exhibited reduced mean and axial diffusivity, increased mean kurtosis, and higher neurite dispersion in deep white matter compared to non-COVID controls. Higher levels of negative affect correlated with increased mean kurtosis and reduced free water in white matter among PACS patients. |
| Dacosta-Aguayo (2024a) | Cross-sectional | N = 53 PCC patients | N/A | MRI | Cognitive deficits including executive function impairments, attention deficits, and memory impairments. Increased radial diffusivity in white matter tracts correlated with memory deficits. Higher fronto-parietal resting-state activity was linked to lower memory performance. Increased connectivity between bilateral hippocampus, right hippocampus & left amygdala, and right hippocampus & left middle temporal gyrus. These connectivity changes were negatively correlated with memory performance, though results did not survive FDR correction |
| Dacosta-Aguayo (2024b) | Cross-sectional | N = 53 PCC patients | N/A | MRI | Cognitive impairment was common, particularly in attention (55%), executive function (59%), and memory (40%) domains. Reduced cortical thickness in the left parahippocampal region and right caudal middle frontal cortex correlated with memory impairment. |
| Danielaa (2022) | Cross-sectional | N = 46 PCC patients | N/A | MRI  (n = 36) | Based on executive function abilities, 19.6% of PCC patients presented with neurocognitive disorder; the only clinical determinant was the requirement for oxygen during acute COVID-19 infection. |
| Deodato  (2024) | Controlled study with an intervention and a control group | N =20 PCC patients  (n = 10 underwent rehabilitation,  n = 10 served as controls) | 20 sessions (1-hour each) of dual-task augmented reality rehabilitation, targeting both cognitive and motor functions | Transcranial Magnetic Stimulation (TMS) to assess cortical excitability | Significant improvement in cognitive performance in the intervention group, shown by reduced TMT-B execution time and higher Frontal Assessment Battery (FAB) scores. No significant differences between groups in fatigue-related measures (FSS, six-minute walking test, handgrip endurance) |
| Diez-Cirarda (2023) | Cross-sectional | N = 122  (n = 36 healthy controls,  n = 29 PCC patients previously hospitalised,  n = 57 PCC patients non-hospitalised) | N/A | fMRI | Greater cognitive deterioration in hospitalised patients, with reduced functional connectivity between left and right parahippocampal areas and increased white matter diffusivity (compared to non-hospitalised patients). |
| Diez-Cirarda (2023) | Cross-sectional | N = 154  (n = 84 PCC patients,  n = 33 healthy controls, n = 37 healthy controls for blood biomarker data) | N/A | MRI & rs-fMRI | Reduced hippocampal volume in PCC patients, compared to controls, correlated with cognitive dysfunction. Lower white matter volume in areas adjacent to the hippocampus and reduced connectivity of the hippocampus with the right parietal area and the left parahippocampal area. |
| Diez-Cirarda  (2024) | Cross-sectional | N = 129 PCC patients | N/A | MRI | Fatigue prevalence in 86% of patients and strongly correlated with subjective cognitive complaints. Fatigue was linked to alterations in frontal, temporal, and cerebellar connectivity. Distinct patterns emerged for mental vs. physical fatigue. White matter diffusivity changes in forceps minor, anterior corona radiata, and anterior cingulum were associated with both fatigue and cognitive complaints. |
| Dressing (2022) | Prospective cohort | N = 76  (n = 31 PCC patients,  n = 45 healthy controls) | N/A | MRI (n = 6) &  PET (n = 14) | PCC patients performed above cut-off on cognitive batteries, although showed some impairment. There was no association between PET findings and cognitive performance, nor significant differences from the control group. |
| Du  (2024) | Cross-sectional | N = 135  (n = 26 with chronic insomnia (without exacerbation),  n = 24 with chronic insomnia (with exacerbation),  n = 40 with no sleep disorder,  n = 30 with new-onset insomnia after Omicron infection,  n = 15 healthy controls who never had Omicron) | N/A | MRI | Cortical thickness changes were similar across Omicron-infected individuals with and without chronic insomnia. Patients with chronic insomnia who worsened post-Omicron had increased medial orbitofrontal cortical thickness and higher proton density values. Patients with new-onset insomnia after Omicron had reduced pericalcarine cortical thickness and lower proton density values. Reduced pericalcarine thickness was correlated with higher anxiety and depression scores. |
| Ferrucci  (2023) | Retrospective | N = 7 PCC patients | N/A | PET | Three patients showed hypometabolic patterns in the left temporal mesial area, some pontine involvement, and patterns in the bilateral prefrontal area with asymmetric parietal impairment. One patient presented with significant Aβ deposition. |
| Furlanis  (2023) | Cross-sectional | N = 20 PCC patients with brain fog | N/A | MRI & EEG | Structural MRI was normal for all patients. EEG showed slowing delta activity in nine patients. Four patients presented with epileptic discharges. |
| Gangemi  (2024) | Longitudinal observational | N = 40  (n = 10 PCC patients with cognitive fog symptoms,  n = 10 PCC patients with cognitive fog and anosmia symptoms,  n = 20 healthy controls) | N/A | EEG | Significant alterations in P300 latency and beta band rhythms were observed in individuals with cognitive fog. These neurophysiological changes persisted 8 months post-recovery. No significant differences were found between individuals with and without anosmia. |
| Gezegen  (2023) | Cross-sectional observational | N =  (n = 36 hyposmic PCC  n = 21 normosmic PCC  n = 25 healthy controls) | N/A | MRI | Hyposmic participants had lower cognitive scores, particularly in language and overall cognitive function. Sniffin’ Sticks test showed poorer discrimination and identification abilities in hyposmic individuals. Decreased OB volumes were observed in hyposmic participants compared to normosmics and healthy controls. Cortical atrophy was found in the left lateral orbitofrontal cortex in hyposmic individuals. A correlation was found between olfactory discrimination/identification scores and structural changes in the OB and left orbital sulci. |
| González-Rosa  (2024) | Retrospective multicenter observational cohort | N = 48  (n = 24 PCC patients,  n = 24 healthy controls) | N/A | MRI | Cognitive assessments were mostly normal, except for executive function impairments in mild COVID-19 patients. Increased juxtacortical white matter hyperintensities observed post-COVID. Thalamic and occipital volume loss detected  Reduced functional connectivity in the left precuneus and cuneus (DMN) and the right angular gyrus and left precuneus (DAN). Reduced thalamic volume was the strongest predictor of executive function impairment. |
| Guedj  (2021) | Retrospective | N = 35 PCC patients  (n = 44 database healthy controls) | N/A | PET | Compared to healthy controls, PCC patients showed hypometabolism in the olfactory gyrus, the right temporal lobe, thalamus, brainstem, and cerebellum. These were significantly associated with functional complaints, and their duration post COVID-19-recovery |
| Guillén  (2024) | Longitudinal observational | N = 46 PCC patients | N/A | MRI | The most affected cognitive domains were attention-executive function and verbal memory. Apathy, moderate-severe anxiety, and severe fatigue were common. Visual memory correlated with total GM and subcortical GM volume, but no evidence of neuronal damage or inflammation markers was found. Cognitive impairment persisted in most participants over time, though fewer exhibited abnormal cognitive evaluations at follow-up. No association was found between cognitive deficits and structural brain abnormalities, elevated cytokines, or neuronal damage markers. |
| Heine  (2023) | Cross-sectional | N = 144  (n = 50 PCC patients,  n = 47 patients with multiple sclerosis and fatigue,  n = 47 healthy controls) | N/A | MRI | PCC fatigue strongly associated with depressive symptoms and sleep quality. PCC patients showed reduction in left thalamus and bilateral putamen. Subcortical changes in multiple sclerosis-related fatigue were associated with lesion load, rather than fatigue score. |
| Hosp  (2024) | Cross-sectional comparative analysis | N = 173  (n = 89 PCC patients  n = 38 PCC unimpaired  n = 46 healthy controls) | N/A | MRI | Widespread changes in cerebral microstructure, likely resulting from a shift in volume from neuronal compartments to free fluid, are linked to the severity of the initial infection. The correlation of these changes with cognitive function, olfaction, and fatigue reveals distinct networks affected, closely aligning with the anatomical and functional basis of these symptoms. |
| Hu  (2024) | Cross-sectional & longitudinal | N = 124 PCC | N/A | MRI | Post-COVID brain fog persists independently of mood, fatigue, or sleepiness and is linked to inflammatory changes rather than neurodegeneration. MRI abnormalities were rare, but cerebrospinal fluid analysis revealed monocyte recruitment, chemokine signaling, and suppressed interferon response in myeloid cells. Slow recovery was associated with increased inflammatory markers (CXCL8, CCL3L1, sTREM2), suggesting a myeloid-driven mechanism. |
| Huang  (2023) | Longitudinal (2 years) | N = 30  (n = 17 PCC patients,  n = 13 healthy controls) | N/A | MRI | Large-scale brain regions showed a trend towards recovery, from 1 to 2 years post COVID-19 infection. However, white matter abnormalities present at 2 years, correlated with cognitive deficits in PCC patients. |
| Hugon  (2022) | Case series | N = 3 PCC patients | N/A | PET | Cognitive decline and cognitive deficits can be associated with hypometabolism in the brainstem (pons). |
| Jin  (2024) | Cross-sectional | N = 92 PCC patients | N/A | MRI | FC patterns predict sleep disturbances post-COVID. MTP-VTA connectivity mediated the link between anxiety and poor sleep. Dorsal raphe connectivity correlated with daytime dysfunction. |
| Joshi  (2024) | Cross-sectional | N = 23  (n = 15 PCC patients,  n = 8 healthy controls) | N/A | MRI | Long COVID patients exhibited increased cortical thickness in the caudal anterior, isthmus, and posterior cingulate gyrus. Higher grey matter volume was observed in the posterior cingulate and isthmus cingulate. Cortical thickness and grey matter volume correlated with disease severity, clinical dementia rating, and anxiety scores. |
| Kamamuta (2023) | Cross-sectional | N = 60  (n = 46 PCC patients,  n = 14 patients with covid vaccine side effects) | 10 sessions, the physician selected the stimulation protocol based on patient symptoms | TMS | No difference in the initial psychiatric symptoms between the two groups. There was significant improvement in anxiety, depression, and other psychology measures in both groups after TMS. |
| Kiatkittikul (2022) | Cross-sectional | N = 13 PCC patients | PET performed at a median time of 32 days after COVID-19 infection | rsfMRI & PET | Patients with multiple PCC symptoms (fatigue, depression, anxiety, loss of taste and smell, cognitive impairment) had multiple hypometabolic areas of the frontal, temporal, parietal, occipital lobes, and the thalamus |
| Klinkhammer (2023) | Prospective cohort | N = 205 PCC patients  (n = 101 previously admitted to ICU  n = 104 not admitted to ICU) | One-hundred-and-one PCC patients were previously admitted to ICU, 104 were not admitted to ICU | MRI | PCC patients who had been admitted to ICU during acute COVID-19, had significantly more microbleeds per patient; more commonly in the corpus callosum. No other differences in MRI abnormalities between the two groups and no relationship between cognitive performance and number of microbleeds. |
| Klinkhammer  (2024) | Prospective, multicenter cohort | N = 205 PCC patients | N/A | N/A |  |
| Klírová  (2024) | Double-blind, randomized, sham-controlled trial | N = 33 PCC patients  (n = 16 in the active group,  n = 17 in the sham group) | 20 tDCS sessions over four weeks  Target area: Prefrontal cortex  Primary outcome: Change in Fatigue Impact Scale (FIS) score  Secondary outcomes: Anxiety, depression, quality of life, and cognitive performance | MRI | No significant difference in fatigue reduction between active and sham groups. No significant intergroup differences in anxiety, depression, quality of life, or cognitive performance. Potential confounders: Small sample size, baseline FIS differences, or suboptimal stimulation parameters. |
| Kurakh  (2024) | Cross-sectional observational | N = 21 PCC patients | N/A | MRI | PCC patients reported difficulties with concentration, memory, and information processing. 15 experienced confusions, and 10 suffered from anxiety. 14 had chronic headaches, 3 dizziness, and 4 experienced both symptoms. MRI imaging revealed varying degrees of neurodegeneration in all cases. |
| Liang (2023) | Cross-sectional | N = 50  (n = 26 PCC patients,  n = 24 healthy controls) | N/A | MRI | No significant difference in cognitive symptoms, between PCC patients and controls. PCC patients experienced more psychiatric symptoms and perceived stress. PCC patients had more restricted diffusivities in white matter tracts. Greater fatigue was related to higher fractional anisotropy bilaterally, in both groups. |
| Manganotti (2023) | Cross-sectional | N = 34  (n = 18 PCC patients with brain fog,  n = 16 healthy controls) | Ten stimuli delivered for each intracortical inhibition protocol in a pseudo-randomised sequence. | TMS | PCC patients performed lower on executive function measures, compared to controls, and showed reduced intracortical inhibition related to GABAb. |
| Martini  (2022) | Cross-sectional | N = 26 acute & subacute PCC patients  (n = 125 database healthy controls) | Acute and subacute PCC patients compared cross-sectionally, with one patient followed up longitudinally (5 months). PCC patients were compared against healthy controls from a database | PET | Most severe brain dysfunction for acute PCC patients, with widespread diffuse cortical hypometabolism. Some functional improvement in the longitudinal case-report, with nearly complete recovery of brain functionality. Findings suggest the cortical functional impairment in PCC might be transient. |
| Miskowiak (2023) | Cross-sectional | N = 14 PCC patients | Eight patients comprised the ‘impaired’ (significantly cognitively impaired) group and 6 the ‘intact’ (cognitively normal) group | PET | Hypermetabolism in the cognitively impaired group correlated with cognitive deficits. Higher metabolism in the cerebellum correlated with the severity of working memory and executive function deficits, in both groups. |
| Muccioli  (2023) | Cross-sectional | N = 49  (n = 23 PCC patients with olfactory dysfunction  n = 26 healthy controls) | Thirteen regions of interest, typically associated with the olfactory network | rs-fMRI | Significant negative correlations between strength of connections in the right thalamus and short-term verbal memory performance, in PCC patients. Global modularity coefficient of the olfactory network positively correlated with performance on the olfactory task in PCC patients. |
| Nagy  (2024) | Experimental, cross-sectional study with cognitive training intervention | N = 50  (n = 30 PCC patients,  n = 20 healthy controls) | Adaptive task-switching training protocol | EEG | Post-COVID older adults showed a shift from global to local neural processing, suggesting accelerated neural aging. Before training, post-COVID females had higher frequency band power and increased entropy compared to healthy controls. Cognitive training improved neural processing only in post-COVID females, reducing the observed differences. |
| Nelson  (2024) | Cross-sectional | N = 91  (n = 56 PCC patients,  n = 35 individuals with normal recovery) | N/A | MRI | Long-COVID patients exhibited lower mean diffusivity in multiple white matter regions, including the internal capsule, corona radiata, corpus callosum, superior fronto-occipital fasciculus, and posterior thalamic radiation. Effect sizes were small and no significant differences were found for other DTI metrics. No significant cognitive differences were detected between long-COVID and normal recovery groups. |
| Niemczak  (2025) | Pilot study | N =20  (n = 10 PCC patients with persistent cognitive fatigue,  n = 10 healthy controls) | N/A | fMRI (2-back memory task) | PASC participants reported greater CF throughout the task. No overall differences in fatigue network activation, but PASC patients recruited more brain regions inside and outside the fatigue network. The PASC group exhibited greater frontal activation, suggesting they exerted more mental effort to perform cognitive tasks. |
| Noda  (2023) | Case series | N = 23 PCC patients (met DSM criteria for depression/ anxiety after COVID infection) | One session of intermittent theta burst stimulation (iTBS) (6 minutes), followed by low frequency repetitive TMS (10 minutes) (20 sessions) | TMS, & iTBS | Significant improvement in depressive symptoms only for males. Significant improvement in subjective and objective depressive symptoms for all patients. Significant improvement in subjective chronic fatigue and cognitive impairment (brain fog). |
| O'Connor  (2024) | Cross-sectional | N = 72  (n = 31 with PCC,  n = 41 asymptomatic no COVID controls) | N/A | MRI | NeuroPASC participants had larger cerebral WM volume, particularly in the prefrontal and anterior temporal regions. Higher mean kurtosis in WM, suggesting ongoing neuroinflammation. NeuroPASC participants performed worse on cognitive tests, with deficits in attention, concentration, verbal learning, and processing speed. Elevated levels of interferon (IFN)-λ1 and IFN-λ2/3 were found in NeuroPASC participants, indicating immune dysregulation. |
| Oliver-Mas (2023) | Randomised, double-blind controlled pilot | N = 47 PCC patients | Twenty-three patients received active treatment, and 24 received sham stimulation. Current was ramped up for 15s, until reaching 2mA and ramped down for 15s. Followed by 20 minutes of constant current at 2mA. In sham condition, current was ramped up and down at the beginning and end of the session, and turned off for the 20-minute session (8 sessions, 8 consecutive days for 2 weeks) | tDCS | Active stimulation associated with statistically significant improvement in physical fatigue immediately after treatment, and after 1 month, compared to sham stimulation. No significant effect for cognitive fatigue and quality of life |
| Ortelli  (2020) | Cross-sectional | N = 12 (PCC patients with fatigue) and matched controls | Stimulation of the right ulnar nerve (wrist) to record compound muscle action potentials. Recorded five motor-evoked potentials (MEPs) from the relaxed first dorsal interosseous muscle. | TMS | PCC patients had smaller baseline peak amplitude of compound muscle potentials that was not affected by a pinching (fatiguing) task. After the task, controls, but not PCC patients, showed a decline in MEPs. |
| Ortelli  (2023) | Cross-sectional | N = 36  (n= 18 PCC patients  n = 18 healthy controls) | N/A | EEG | Normal global cognitive scores in PCC patients- no clinical cognitive impairment. Bilateral distribution of reduction in brain source activity in delta band; hypothesise dysfunction in the GABAergic system and some involvement in the pathophysiology of brain fog in PCC. |
| Ortelli  (2022) | Cross-sectional | N = 89  (n = 67 PCC patients  n = 22 healthy controls) | N/A | TMS | No significant difference in the maximum force exerted on a pinching task between PCC patients and controls. PCC patients showed reduced motor cortex excitability and lower motor output; they had higher resting motor thresholds and lower motor evoked potentials. |
| Ortelli, Quercia (2023) | Cross-sectional | N = 36  (n = 18 PCC patients,  n = 18 healthy controls) | N/A | EEG | PCC patients had lower delta activity at rest, in frontal, parietal and temporal regions, commonly associated with executive processes. PCC patients presented with fatigue and cognitive complaints, without clinically significant cognitive impairment. |
| Pendolino  (2023) | Retrospective | N = 100 PCC with anosmia issues | N/A | N/A | 20% of patients recovered normal olfactory function. Dysosmic patients had lower quality-of-life scores in energy/fatigue and emotional well-being. Higher nasal airflow (PNIF) was associated with better odor threshold, while allergy sensitivity (SPT) negatively impacted odor identification. Persistent smell loss significantly affected quality of life, but recovery improved it to normal levels. |
| Perlaki  (2023) | Prospective case-control | N = 55  (n = 38 PCC patients,  n = 37 healthy controls) | N/A | MRI | PCC patients showed lower bilateral cortical thickness, reduced subcortical grey matter volume, and smaller right olfactory bulb volume compared to healthy controls. Individuals with moderate to severe anosmia had significantly lower cortical thickness bilaterally. Localized reductions in cortical thickness were observed in the right lateral orbitofrontal cortex |
| Petersen  (2024) | Longitudinal cohort | N = 233 PCC | N/A | MRI | Olfactory dysfunction declined over time, reported by 67.1% during acute infection, 21.0% at baseline, and 17.5% at follow-up. Individuals with post-acute olfactory dysfunction had significantly lower olfactory bulb volume at baseline. Olfactory bulb volume at baseline predicted future olfactory function, but was not associated with cognitive test performance. |
| Rothstein (2023) | Cross-sectional | N = 24 PCC  (compared against normative database) | N/A | MRI | Diminished gray matter volume in PCC patients, with larger-than-expected volumes in the hippocampus and thalamus. |
| Rua  (2024) | Cross-sectional study | N = 81  (n = 30 PCC patients,  n = 51 healthy controls) | N/A | MRI | Post-COVID patients exhibited increased MR susceptibility in the brainstem, particularly in the medulla, pons, and midbrain. Higher susceptibility in the inferior medullary reticular formation and raphe nuclei correlated with:  Worse acute disease severity, higher inflammatory markers during acute infection, poorer functional recovery. |
| Ruzicka  (2024) | Cross-sectional observational | N = 213 PCC patients  (n = 55 were further evaluated by means of brain imaging  n = 78 underwent neurocognitive testing) | N/A | MRI | Cognitive symptoms were reported by 73.3% of PCC patients, affecting working memory, attention, and concentration. Brain imaging abnormalities were found in 14.5% but were likely unrelated to PCC. While symptom severity did not impact neurocognitive performance, those with severe symptoms had higher rates of depression, fatigue, sleep disturbances, and psychiatric conditions. |
| Saleh (2021) | Comparative single evaluation cross-sectional | N = 144  (n = 106 PCC patients,  n = 38 healthy controls) | Simple reaction (processing speed) and forced choice tasks (visual sensitivity and visual attention) on a computer screen | EEG | PCC patients who recovered from severe COVID-19 has slower reaction times, compared to never infected controls. Patients recovered from mild-moderate COVID-19 showed similar performance to never-infected controls. |
| Santana (2023) | Prospective double-blind, randomised, sham controlled clinical trial | N = 70 PCC patients with fatigue | Personalised rehabilitation programme for PCC-related fatigue and educational material. Neurostimulation: 30s ramp up, held at 3mA for 30 minutes and 30s ramp down (10 sessions). | tDCS | Significantly greater reduction in fatigue (cognitive and psychosocial measures) for the active condition, but no difference in physical fatigue. Significant reduction in anxiety, and quality of life for the active condition. No significant difference in pain between groups. |
| Sasaki (2023) | Cross-sectional | N = 12 PCC patients with chronic fatigue and cognitive dysfunction | Ten consecutive stimulations, once every two weeks, 10Hz stimulation (for approximately 78 days) | rTMS | TMS improved PCC-related fatigue and cognitive symptoms. There was significant improvement in measures of intelligence (IQ) after TMS. |
| Serrano Del Pueblo (2024) | Cross-sectional | N = 105  (n = 83, PCC patients  n = 22 healthy controls) | N/A | MRI |  |
| Sklinda (2021) | Cross-sectional | N = 23  (n = 12, PCC patients with brain fog  n = 11 healthy controls) | N/A | MRI | Changes in brain metabolites in PCC patients, without any other radiological symptoms of cerebral injury. Increased concentration of glutamate and glutamine in PCC patients and mild reduction of lactic acid in the deep gray matter. |
| Thomasson (2023) | Cross-sectional | N = 105 PCC patients (compared against healthy controls from a different study) | N/A | rs-fMRI | Neuropsychological PCC symptoms not associated with emotion recognition ability. Performance on verbal and episodic memory tasks was the best predictor of multimodal emotion recognition in PCC patients. |
| Vakani  (2025) | Cross-sectional | N = 43 working-age adults with a history of COVID-19 | N/A | MRI | Higher persistent COVID-19 symptom load correlated with smaller putamen volume. Reduced putamen volume was associated with lower accuracy on working memory, executive function, and recognition memory tasks, as well as longer response times for executive function. Poorer mental health and sleep quality were linked to greater symptom load. Putamen volume fully mediated the relationship between persistent COVID-19 symptoms and executive function impairment. |
| Versace (2021) | Cross-sectional | N = 20  (n = 10 PCC patients  n = 10 healthy controls) | N/A | TMS | Reduced inhibition in the motor cortex, evidenced by a disruption of GABAa and GABAb, mediated by short and long interval intracortical inhibition, for PCC patients. |
| Versace (2023) | Double blind placebo controlled randomised controlled trial | N = 34 PCC patients | Seventeen atients were administered palmitoylethanolamide co-ultramicronized with flavounoid luteolin (PEA-LUT), for 8 weeks | TMS | Oral administration of PEA-LUT for 8 weeks, increased the GABAergic activity of the motor cortex in PCC patients with long-term fatigue and improved long term potentiation-like cortical plasticity. |
| Voruz (2022) | Cross-sectional | N = 102 PCC patients (divided based on anosognosia) | N/A | MRI & fMRI | No structural differences between anosognosic and nosognosic patients. Anosognosic patients had greater memory impairment, fewer self-reported psychiatric symptoms (depression, stress, anxiety), better self-reported quality of life, reduced connectivity between the default mode network. |
| Wojcik (2023) | Cross-sectional | N = 120 (  n = 80 PCC patients,  n = 40 healthy controls) | Forty PCC patients had serious brain fog, and 40 did not have brain fog. Three task paradigms: face recognition, digit span, task switching | EEG | Significant difference in cortical activity (measured as event related potentials) of patients with brain fog, compared to those without brain fog and healthy controls. |
