# Supplementary material for: Cognitive and Psychological Symptoms in Post-COVID-19 Condition: A Systematic Review of Structural and Functional Neuroimaging, Neurophysiology, and Intervention Studies
Source: Arch Rehabil Res Clin Transl. 2025 May 9;7(3):100461. doi: 10.1016/j.arrct.2025.100461 (PMC12447218; doi:10.1016/j.arrct.2025.100461)
Supplement: Supplementary file 2 [file mmc2.docx]

Table S1. *Risk of bias in individual randomised studies, based on the Critical Appraisals Skills Programme (CASP) checklist for randomised controlled trials*

| Domain | Chang (2023) | Oliver-Mas (2023) | Santana (2023) | Versace (2023) | Klírová  (2024) |
| --- | --- | --- | --- | --- | --- |
| Did the trial address a clearly focused issue? | + | + | + | + | + |
| Was the assignment of patients randomised? | + | + | + | ? | + |
| Were all participants who entered the study accounted for at its conclusion? | + | + | + | + | + |
| Were the patients, health workers and study personnel ‘blind’ to treatment? | - | + | + | ? | + |
| Were the groups similar at the start of the trial? | + | + | + | + | - |
| Aside from experimental intervention, were the groups treated equally? | ? | ? | + | ? | + |
| Can the results be applied in your local population or context? | + | + | + | + | ? |
| Were all clinically important outcomes considered? | ? | ? | ? | ? | - |
| Are the benefits worth the harm and costs? | + | ? | + | ? | ? |

*Note.* The above domains were assessed for each study and risk of bias was judged as high (-), unclear (?) or low (+).

Table S2. *Risk of bias in individual cohort studies, based on the Critical Appraisals Skills Programme (CASP) checklist for cohort studies*

| Domain | Ceccheti (2022) | Dressing (2022) | Huang (2023) | Ferrucci (2023) | | Guedj (2021) | Klinkhammer (2023 & 2024) | | González-Rosa  (2024) | Petersen  (2024) |
| --- | --- | --- | --- | --- | --- | --- | --- | --- | --- | --- |
| Did the study address a clearly focused issue? | + | + | + | + | + | | | + | + | + |
| Was the cohort recruited in an acceptable way? | + | + | + | + | + | | | + | + | + |
| Was the exposure accurately measured to minimise bias? | ? | ? | + | + | - | | | + | + | + |
| Was the outcome accurately measured to minimise bias? | + | - | + | + | + | | | + | + | + |
| Have the authors identified all important confounding factors? | ? | ? | ? | + | + | | | + | - | + |
| Have they taken account of the confounding factors in the design and/or analysis? | - | - | + | + | ? | | | + | ? | + |
| Was the follow up of subjects complete enough? | + | + | + | + | + | | | ? | + | + |
| Was the follow up of subjects long enough? | ? | ? | + | ? | ? | | | + | + | + |
| Did you believe the results? | + | ? | + | + | + | | | + | + | + |
| Can the results be applied to the local population? | ? | + | + | + | + | | | + | ? | + |
| Do the results of this study fit with other available evidence? | + | - | ? | + | + | | | + | + | + |
| What are the implications of this study for practice? | + | + | + | + | + | | | ? | ? | + |

*Note.* The above domains were assessed for each study and risk of bias was judged as high (-), unlcear (?) or low (+).

Table S3. *Risk of bias (RoB) for quantitative, cohort studies evaluated with the Newcastle–Ottawa Scale (NOS)*

| Study | Selection (0–5) | Comparability (0–2) | Outcome (0–3) |
| --- | --- | --- | --- |
| Ajcevic (2023) | ★★★ | ★★ | ★★ |
| Andriuta (2022) | ★★★ | ★★ | ★★ |
| Besteher (2022) | ★★★ | ★★ | ★★ |
| Besteher (2023) | ★★★ | ★★ | ★★ |
| Bowen (2023) | ★★★ | ★ | ★★ |
| Churchill (2023) | ★★★ | ★ | ★★★ |
| Diez-Cirarda (2023) | ★★ | ★★ | ★★ |
| Diez-Cirarda, Yus (2023) | ★★★ | ★★ | ★★ |
| Furlanis (2023) | ★★★ | ★★ | ★★ |
| Heine (2023) | ★★★ | ★★ | ★★★ |
| Kamamuta (2023) | ★★★ | ★★ | ★★★ |
| Kiatkittikul (2021) | ★★ | ★ | ★★★ |
| Liang (2023) | ★★★ | ★★ | ★★★ |
| Manganotti (2023) | ★★★ | ★★ | ★★ |
| Martini (2022) | ★★ | ★ | ★★ |
| Miskowiak (2022) | ★★★ | ★★ | ★★ |
| Muccioli (2023) | ★★★ | ★★ | ★★ |
| Ortelli (2020) | ★★ | ★★ | ★★ |
| Ortelli (2022) | ★★ | ★ | ★★ |
| Ortelli (2023) | ★★★ | ★★ | ★★★ |
| Rothstein (2023) | ★★ | ★★ | ★★ |
| Saleh (2021) | ★★★ | ★ | ★ |
| Santoyo-Mora (2022) | ★★★ | ★★ | ★★ |
| Sasaki (2023) | ★★★ | ★★ | ★★ |
| Sklinda (2021) | ★★ | ★★ | ★★ |
| Thomasson (2023) | ★★★ | ★★ | ★★ |
| Versace (2021) | ★★ | ★★ | ★★ |
| Voruz (2022) | ★★ | ★★ | ★★ |
| Wojak (2023) | ★★★ | ★ | ★★ |
| Serrano Del Pueblo (2024) | ★★★★ | ★★ | ★★★ |
| Hosp (2024) | ★★★★★ | ★★ | ★★★ |
| Kurakh (2024) | ★★★ | ★ | ★★ |
| Ruzicka (2024) | ★★★★ | ★ | ★★★ |
| Hu (2024) | ★★★★★ | ★★ | ★★★ |
| Pendolino (2023) | ★★★ | ★ | ★★ |
| Gezegen (2023) | ★★★★ | ★★ | ★★★ |
| Vakani (2025) | ★★★★ | ★★ | ★★★ |
| Cataldo (2024) | ★★★★★ | ★★ | ★★★ |
| Churchill (2024) | ★★★★ | ★★ | ★★★ |
| Besteher (2024) | ★★★★★ | ★★ | ★★★ |
| Nelson (2024) | ★★★★★ | ★★ | ★★★ |
| Deodato (2024) | ★★★ | ★ | ★★ |
| Gangemi (2024) | ★★★★ | ★★ | ★★★ |
| O’Connor (2024) | ★★★★★ | ★★ | ★★★ |
| Du (2024) | ★★★★ | ★★ | ★★★ |
| Dacosta-Aguayo (2024, Frontiers in Neurology) | ★★★★★ | ★★ | ★★★ |
| Diez-Cirarda (2024) | ★★★★ | ★★ | ★★★ |
| Baker (2023) | ★★★★★ | ★★ | ★★★ |
| Niemczak (2025) | ★★★ | ★ | ★★ |
| Alkhormani (2024) | ★★★★ | ★ | ★★ |
| Rua (2024) | ★★★★★ | ★★ | ★★★ |
| Dacosta-Aguayo (2024, AJNR) | ★★★★★ | ★★ | ★★★ |
| Casula (2024) | ★★★★ | ★★ | ★★★ |
| Babiloni (2024) | ★★★★ | ★★ | ★★★ |
| Jin (2024) | ★★★★ | ★★ | ★★★ |
| Nagy (2024) | ★★★★ | ★★ | ★★★ |
| Joshi (2024) | ★★★ | ★ | ★★ |

*Note.* A greater number of stars indicates greater study quality for each domain (selection, comparability, outcome)

Table S4. *Risk of bias (RoB) for quantitative, case-series evaluated with Quality Appraisal Checklist for Case Series studies*

| Domain | Hugon (2022) | Noda (2023) |
| --- | --- | --- |
| Was the hypothesis/ aim/ objectives of the study clearly stated? | ? | + |
| Was the study conducted prospectively? | - | + |
| Were the cases collected in more than one centre? | - | - |
| Were patients recruited consecutively? | ? | ? |
| Were the characteristics of the patients included in the study described? | + | + |
| Were the eligibility criteria (i.e. inclusion and exclusion criteria) for entry into the study clearly stated? | - | + |
| Did the patients enter the study at a similar point in the disease? | ? | ? |
| Was the intervention of interest clearly described? | + | + |
| Were relevant outcome measures described a priori? | + | + |
| Were outcome assessors blinded to the intervention that patients received? | ? | ? |
| Were the relevant outcomes measured using appropriate objective/ subjective methods? | + | + |
| Were the relevant outcome measures made before and after the intervention? | - | + |
| Were the statistical tests used to assess the relevant outcomes appropriate? | + | + |
| Was follow-up long enough for important events and outcomes to occur? | ? | ? |
| Were losses to follow-up reported? | + | + |
| Did the study provide estimates of random variability in the data analysis of relevant outcomes | - | - |
| Were the adverse events reported? | - | + |
| Were the conclusions of the study supported by results? | + | + |
| Were both competing interests and sources of support for the study reported? | + | + |

*Note.* The above domains were assessed for each study and risk of bias was judged as high (-), unclear (?) or low (+).

Strategy for systematic search

PubMed

- “Long covid” [text word]
- Long covid
- “post-acute COVID-19 syndrome”
- Post-COVID syndrome [MeSH]
- “post-COVID syndrome” [text word]

Combine with OR

**(((("long covid"[Text Word]) OR (long covid)) OR ("post-acute COVID-19 syndrome")) OR (post-COVID syndrome[MeSH])) OR ("post-COVID syndrome"[Text Word])**

- EEG [text word]
- Electroencephalogr*
- Electroencephalogram[MeSH]
- MRI [text word]
- “magnetic resonance imaging” [MeSH]
- magnetic resonance imag*[text word]
- fMRI [text word]
- functional magnetic resonance imaging [MeSH]
- DTI [Text word]
- Diffusion tensor imaging [MeSH]
- diffusion tensor imag* [text word]
- Fiber tractography [text word]
- diffusion MRI [text word]
- TMS [text word]
- Transcranial magnetic stimulation [MeSH]
- tDCS [text word]
- transcranial direct current stimulation [MeSH]
- transcranial alternative current stimulation [text word]
- neuroimaging [MeSH]
- neurostim* [text word]

Combine with OR

**(EEG[Text Word]) OR (electroencephalogr*) OR (electroencephalogram[MeSH Terms]) OR (MRI[Text Word]) OR (magnetic resonance imaging[MeSH Terms]) OR (magnetic resonance imag*[text word]) OR (fmri[Text Word]) OR (functional magnetic resonance imaging [MeSH Terms]) OR (DTI[Text Word]) OR (diffusion tensor imaging[MeSH Terms]) OR (diffusion tensor imag*[text word]) OR (diffusion MRI[text word]) OR (TMS[Text Word]) OR (transcranial magnetic stimulation[MeSH Terms]) OR (tDCS[Text Word]) OR (transcranial direct current stimulation[MeSH Terms]) OR (tACS[Text Word]) OR (transcranial alternative current stimulation[text word]) OR (neuroimaging[MeSH Terms]) OR (fiber tractography[text word) OR (neurostim*[text word])**

- Neurological rehabilitation [MeSH]
- Cognitive training [text word]
- Cognitive training [MeSH]
- Brain training [MeSH]
- Cost of illness [MeSH]
- Disease burden [MeSH]
- Quality of life [MeSH]
- Quality of life [text word]
- Neuropsych*
- Disease burden [text word]
- Cognitive dysfunct*
- Cognitive dysfunction [MeSH]
- Cognitive problem* [text word]
- Cognitive defic* [text word]
- Cognitive difficult* [text word]
- Cognitive rehab* [text word]
- Mental* impair* [text word]
- Behavioral symptom [MeSH]
- behavioural symptom*[text word]
- Behavioral symptom*[text word]

Combine with OR

**(cognitive rehab*[text word]) OR (neurological rehabilitation[MeSH Terms]) OR (cognitive training[Text Word]) OR (brain training[MeSH Terms]) OR (cognitive training[MeSH Terms]) OR (cost of illness[MeSH Terms]) OR (disease burden[MeSH Terms]) OR (quality of life[MeSH Terms]) OR (neuropsych*) OR (quality of life[Text Word]) OR (disease burden[Text Word]) OR (cognitive dysfunct*) OR (mental impairment [text word]) OR (cognitive dysfunction[MeSH Terms]) OR (behavioral symptom[MeSH Terms]) OR (cognitive problem*[text word]) OR (cognitive defic*[text word]) OR (cognitive difficult*[text word]) OR (behavioural symptom*[text word]) OR (behavioral symptom*[text word])**

**Search strategy**

**(((((("long covid"[Text Word]) OR (long covid)) OR ("post-acute COVID-19 syndrome")) OR (post-COVID syndrome[MeSH])) OR ("post-COVID syndrome"[Text Word])) AND ((EEG[Text Word]) OR (electroencephalogr*) OR (electroencephalogram[MeSH Terms]) OR (MRI[Text Word]) OR (magnetic resonance imaging[MeSH Terms]) OR (magnetic resonance imag*[text word]) OR (fmri[Text Word]) OR (functional magnetic resonance imaging [MeSH Terms]) OR (DTI[Text Word]) OR (diffusion tensor imaging[MeSH Terms]) OR (diffusion tensor imag*[text word]) OR (diffusion MRI[text word]) OR (TMS[Text Word]) OR (transcranial magnetic stimulation[MeSH Terms]) OR (tDCS[Text Word]) OR (transcranial direct current stimulation[MeSH Terms]) OR (tACS[Text Word]) OR (transcranial alternative current stimulation[text word]) OR (neuroimaging[MeSH Terms]) OR (fiber tractography[text word) OR (neurostim*[text word]))) AND ((cognitive rehab*[text word]) OR (neurological rehabilitation[MeSH Terms]) OR (cognitive training[Text Word]) OR (brain training[MeSH Terms]) OR (cognitive training[MeSH Terms]) OR (cost of illness[MeSH Terms]) OR (disease burden[MeSH Terms]) OR (quality of life[MeSH Terms]) OR (neuropsych*) OR (quality of life[Text Word]) OR (disease burden[Text Word]) OR (cognitive dysfunct*) OR (mental impairment [text word]) OR (cognitive dysfunction[MeSH Terms]) OR (behavioral symptom[MeSH Terms]) OR (cognitive problem*[text word]) OR (cognitive defic*[text word]) OR (cognitive difficult*[text word]) OR (behavioural symptom*[text word]) OR (behavioral symptom*[text word]))**

EMBASE

A.

'long covid' OR 'long covid'/exp OR 'post-acute covid-19 syndrome' OR 'post-covid syndrome'/exp OR 'post-covid syndrome'

B.

eeg OR electroencephalogr* OR 'electroencephalogram'/exp OR mri OR 'magnetic resonance imaging'/exp OR (magnetic AND resonance AND imag*) OR fmri OR 'functional magnetic resonance imaging'/exp OR dti OR 'diffusion tensor imaging'/exp OR (diffusion AND tensor AND imag*) OR 'fiber tractography' OR 'diffusion mri'/exp OR tms OR 'transcranial magnetic stimulation'/exp OR tdcs OR 'transcranial direct current stimulation'/exp OR (transcranial AND alternative AND current AND stimulation) OR 'neuroimaging'/exp OR neurostim*

C.

'neurorehabilitation'/exp OR 'cognitive training' OR 'cognitive training'/exp OR 'brain training'/exp OR 'cost of illness'/exp OR 'disease burden'/exp OR 'quality of life'/exp OR 'quality of life' OR neuropsych* OR 'disease burden' OR 'cognitive dysfunct*' OR 'cognitive dysfunction'/exp OR 'cognitive problem' OR 'cognitive defic*' OR 'cognitive difficult*' OR 'cognitive rehab*' OR 'mental* impair*' OR 'behavioural symptom*' OR 'behavioral symptom*'

Search strategy

('long covid' OR 'long covid'/exp OR 'post-acute covid-19 syndrome' OR 'post-covid syndrome'/exp OR 'post-covid syndrome') AND (eeg OR electroencephalogr* OR 'electroencephalogram'/exp OR mri OR 'magnetic resonance imaging'/exp OR (magnetic AND resonance AND imag*) OR fmri OR 'functional magnetic resonance imaging'/exp OR dti OR 'diffusion tensor imaging'/exp OR (diffusion AND tensor AND imag*) OR 'fiber tractography' OR 'diffusion mri'/exp OR tms OR 'transcranial magnetic stimulation'/exp OR tdcs OR 'transcranial direct current stimulation'/exp OR (transcranial AND alternative AND current AND stimulation) OR 'neuroimaging'/exp OR neurostim*) AND ('neurorehabilitation'/exp OR 'cognitive training' OR 'cognitive training'/exp OR 'brain training'/exp OR 'cost of illness'/exp OR 'disease burden'/exp OR 'quality of life'/exp OR 'quality of life' OR neuropsych* OR 'disease burden' OR 'cognitive dysfunct*' OR 'cognitive dysfunction'/exp OR 'cognitive problem' OR 'cognitive defic*' OR 'cognitive difficult*' OR 'cognitive rehab*' OR 'mental* impair*' OR 'behavioural symptom*' OR 'behavioral symptom*')

Web of knowledge

((((TS=(long covid)) OR TI=(long covid)) OR TS=(post-acute COVID-19 syndrome)) OR TS=(post-COVID syndrome)) OR TI=(post-COVID syndrome)and Preprint Citation Index (Exclude – Database)

(((((((((((((((((((TI=(EEG)) OR TS=(electroencephalogr*)) OR TS=(electroencephalogram)) OR TS=(MRI)) OR TS=(magnetic resonance imaging)) OR TS=(magnetic resonance imag*)) OR TS=(fMRI)) OR TS=(functional resonance imaging)) OR TS=(DTI)) OR TS=(diffusion tensor imaging)) OR TS=(diffusion tensor imag*)) OR TS=(fiber tractography)) OR TS=(diffusion MRI)) OR TS=(TMS)) OR TS=(transcranial magnetic stimulation)) OR TS=(tDCS)) OR TS=(transcranial direct magnetic stimulation)) OR TS=(transcranial alternative current stimulation)) OR TS=(neuroimag*)) OR TS=(neurostim*) and Preprint Citation Index (Exclude – Database)

(((((((((((((((((((TS=(neurological rehabilitation)) OR TS=(cognitive training)) OR TS=(cognitive training)) OR TS=(brain training)) OR TS=(cost of illness)) OR TS=(disease burden)) OR TS=(quality of life)) OR TS=(quality of life)) OR TS=(neuropsych*)) OR TI=(disease burden)) OR TS=(cognitive dysfunct*)) OR TS=(cognitive dysfunction)) OR TS=(cognitive problem)) OR TS=(cognitive defic*)) OR TS=(cognitive difficult*)) OR TS=(cognitive rehab*)) OR TS=(mental* impair*)) OR TS=(behavioural symptom)) OR TS=(behavioral symptom*)) OR TS=(behavioural symptom*) and Preprint Citation Index(Exclude – Database)
